# A non-dividing cell population with high pyruvate dehydrogenase kinase activity regulates metabolic heterogeneity and tumorigenesis in the intestine

**DOI:** 10.1038/s41467-022-29085-y

**Published:** 2022-03-21

**Authors:** Carlos Sebastian, Christina Ferrer, Maria Serra, Jee-Eun Choi, Nadia Ducano, Alessia Mira, Manasvi S. Shah, Sylwia A. Stopka, Andrew J. Perciaccante, Claudio Isella, Daniel Moya-Rull, Marianela Vara-Messler, Silvia Giordano, Elena Maldi, Niyati Desai, Diane E. Capen, Enzo Medico, Murat Cetinbas, Ruslan I. Sadreyev, Dennis Brown, Miguel N. Rivera, Anna Sapino, David T. Breault, Nathalie Y. R. Agar, Raul Mostoslavsky

**Affiliations:** 1 Candiolo Cancer Institute-FPO, IRCCS, Candiolo, 10060 Italy; 2 Departament de Biologia Cel.lular, Fisiologia i Immunologia, Facultad de Biologia, Universitat de Barcelona (UB), 08028 Barcelona, Spain; 3 Institut de Biomedicina de la Universitat de Barcelona (IBUB), 08028 Barcelona, Spain; 4 The Massachusetts General Hospital Cancer Center, Harvard Medical School, Boston, MA 02114 USA; 5 The MGH Center for Regenerative Medicine, Harvard Medical School, Boston, MA 02114 USA; 6 Division of Endocrinology, Boston Children’s Hospital, Boston, MA 02115 USA; 7 Department of Neurosurgery, Brigham and Women’s Hospital, Harvard Medical School, Boston, MA USA; 8 Department of Radiology, Brigham and Women’s Hospital, Harvard Medical School, Boston, MA USA; 9 University of Georgia, Athens, GA USA; 10 Department of Oncology, University of Torino, Turin, Italy; 11 Department of Pathology, Massachusetts General Hospital and Harvard Medical School, Boston, MA 02114 USA; 12 Center for Systems Biology, Massachusetts General Hospital and Harvard Medical School, Boston, MA 02114 USA; 13 Program in Membrane Biology and Division of Nephrology, Massachusetts General Hospital and Harvard Medical School, Boston, MA 02114 USA; 14 Department of Medical Sciences, University of Torino, Turin, Italy; 15 Department of Pediatrics, Harvard Medical School, Boston, MA 02115 USA; 16 Harvard Stem Cell Institute, Harvard University, Cambridge, MA 02138 USA; 17 Department of Cancer Biology, Dana Farber Cancer Institute, Boston, MA 02115 USA; 18 The Broad Institute of Harvard and MIT, Cambridge, MA 02142 USA; 19 Present Address: Pole of Pharmacology and Therapeutics (FATH), Institut de Recherche Experimentale et Clinique (IREC), UCLeuven, Brussels, Belgium

**Keywords:** Cancer metabolism, Cancer stem cells

## Abstract

Although reprogramming of cellular metabolism is a hallmark of cancer, little is known about how metabolic reprogramming contributes to early stages of transformation. Here, we show that the histone deacetylase SIRT6 regulates tumor initiation during intestinal cancer by controlling glucose metabolism. Loss of SIRT6 results in an increase in the number of intestinal stem cells (ISCs), which translates into enhanced tumor initiating potential in APC^min^ mice. By tracking down the connection between glucose metabolism and tumor initiation, we find a metabolic compartmentalization within the intestinal epithelium and adenomas, where a rare population of cells exhibit features of Warburg-like metabolism characterized by high pyruvate dehydrogenase kinase (PDK) activity. Our results show that these cells are quiescent cells expressing +4 ISCs and enteroendocrine markers. Active glycolysis in these cells suppresses ROS accumulation and enhances their stem cell and tumorigenic potential. Our studies reveal that aerobic glycolysis represents a heterogeneous feature of cancer, and indicate that this metabolic adaptation can occur in non-dividing cells, suggesting a role for the Warburg effect beyond biomass production in tumors.

## Abstract (web summary)

Metabolic reprogramming upon SIRT6 loss induces tumour formation in the intestine but the mechanism is unclear. Here, the authors show that loss of SIRT6 leads to the expansion of epithelial cells with high pyruvate dehydrogenase kinase activity resulting in enhanced stem cell activity and tumour-initiating potential

## Introduction

Reprogramming of core cellular metabolic pathways represents a key feature of most cancer cells. Research done during the last decade has demonstrated that these metabolic adaptations are strictly required for tumor growth and progression^1^, and thus, metabolic reprogramming has been recently upgraded to be a hallmark of cancer^2^. Despite the many efforts made to elucidate the metabolic adaptations of cancer cells and their contribution to tumor progression, surprisingly little is known about the role of metabolic reprogramming during early stages of transformation and the specific metabolic properties of tumor initiating cells (TICs). In this context, identification of metabolic pathways controlling the fate of these cells will help in designing new therapeutic approaches, as TICs have been proposed to drive tumor recurrence and metastatic dissemination. We previously described the NAD^+^-dependent protein deacylase SIRT6 as a potent tumor suppressor in the intestine by controlling glucose metabolism^3^. Loss of this chromatin factor enforces a Warburg-like metabolic reprogramming that is sufficient to drive tumor formation and growth in *APC*^*min*^ mice^3^, suggesting a role for glucose metabolism in the initiation of intestinal and colorectal cancers. Recent work has demonstrated that intestinal stem cells (ISCs) are the cell of origin of most intestinal tumors^4–8^ and, importantly, despite some controversy on the specific metabolic properties of ISCs, it seems clear that increased glucose metabolism is required for ISC function and proliferation^9–11^. However, whether different ISC subtypes exhibit distinct metabolic properties, the underlying molecular mechanisms driving the metabolic control of ISC fate and its putative role on tumorigenesis remain largely unexplored.

Here, we demonstrate that lack of SIRT6 increases the number and activity of ISCs, which translates into an enhanced tumor initiating potential. Importantly, this phenotype is reversed by inhibiting glycolysis, indicating that enhanced glycolytic metabolism in the absence of SIRT6 drives intestinal tumorigenesis by increasing the number of TICs. Furthermore, we uncover the presence of metabolic heterogeneity among intestinal epithelial cells and intestinal adenomas, where we find a cell type with high pyruvate dehydrogenase kinase (PDK) activity (and hence likely glycolytic) exhibiting +4 ISCs and enteroendocrine (EE) cells markers. These cells are quiescent and display stem cell potential, which depends on active glucose metabolism to decrease oxidative metabolism and ROS production. Our results provide an insight into the role of metabolism in driving normal ISCs and early stages of intestinal cancer.

## Results

### Sirt6 controls stem cell activity in the intestine and tumors of *APC*^*min*^ mice by modulating glucose metabolism

**Fig. 1:**
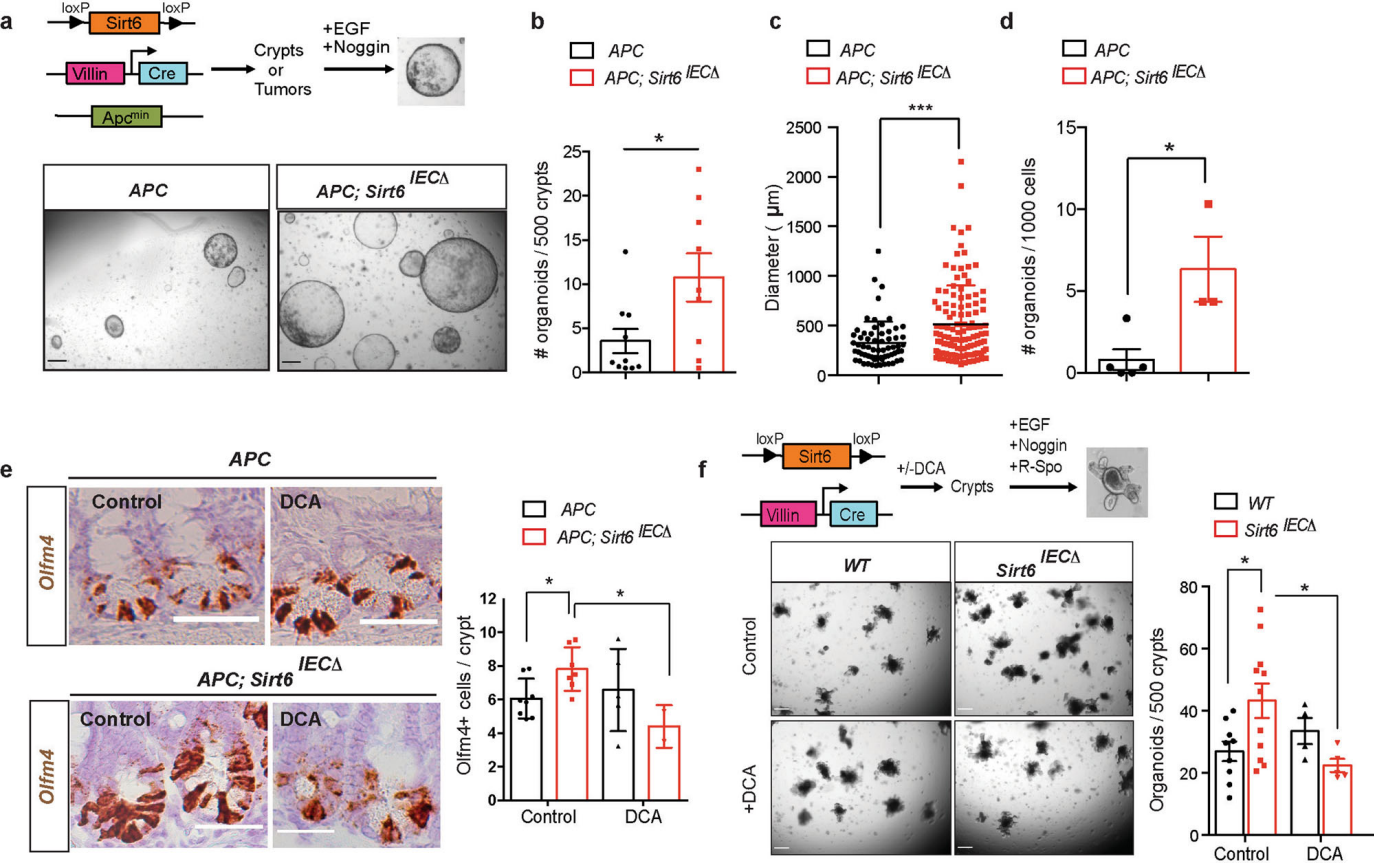
SIRT6-dependent metabolic reprogramming regulates tumor initiation in the intestine. **a** Tumor initiating potential was assayed by organoid formation experiments in EN medium. The pictures illustrate adenoma-derived organoids from *APC* and *APC; Sirt6*^*IECΔ*^ mice. Scale bars, 300 μm. **b** Quantification of number of organoids derived from *APC* (*n* = 10) and *APC; Sirt6*^*IECΔ*^ (*n* = 9) mice (*p* = 0.0263). Each experiment was done in triplicate and data is presented as mean ± SEM. **c** Organoid size was quantified by averaging two diameter measures in organoids derived from *APC* and *APC; Sirt6*^*IECΔ*^ (*n* = 2) mice. The average size of all organoids is represented as mean ± SD (*p* = 0.003). A total of 64 organoids from *APC* mice and 129 from *APC; Sirt6*^*IEC*^ mice were analyzed. **d** Number of organoids derived from adenomas from *APC* (*n* = 5) and *APC; Sirt6*^*IECΔ*^ (*n* = 3) mice (*p* = 0.017). Each experiment was done in triplicate and data is presented as mean ± SD. **e** Representative images of ISH for Olfm4 (left) and quantification of Olfm4+ cells in *APC* and *APC; Sirt6*^*IECΔ*^ untreated (*n* = 8 and *n* = 7, respectively) and treated with DCA (*n* = 5 and *n* = 3, respectively). *P* = 0.0169 (*APC* compared to *APC; Sirt6*^*IECΔ*^) and *p* = 0.0135 (*APC; Sirt6*^*IECΔ*^ control compared to DCA-treated). Data is presented as mean ± SD. Scale bars, 100 μm. **f** ISC activity was measured by organoid formation assays in ENR medium. Left, representative images of 7-day organoids from control and *Sirt6*^*IECΔ*^ mice (untreated and treated with DCA). Right, quantification of organoid number from control and *Sirt6*^*IECΔ*^ mice untreated (*n* = 9 and *n* = 11, respectively) and treated with DCA (*n* = 4 and *n* = 5, respectively). *P* = 0.0273 (*WT* compared to *Sirt6*^*IECΔ*^) and *p* = 0.0279 (*Sirt6*^*IECΔ*^ control compared to DCA-treated). Each experiment was done in triplicate and data is presented as mean ± SEM. Scale bars, 300 μm Two tailed *t*-test (for comparisons between two groups) or one-way ANOVA (for comparisons of more than two groups) were used to determine statistical significance between groups (*p < 0.05; **p < 0.01; ***p < 0.001). Source data are provided as a Source Data file.

Our previous work demonstrated that SIRT6 loss leads to metabolic reprogramming in the intestinal epithelium of *APC*^*min*^ mice, driving an increased number of intestinal adenomas, which were larger in size and exhibited more aggressive features^3^. Prompted by these results, we hypothesized that SIRT6 could have a prominent role in regulating early stage transformation, namely, it may influence TICs. To test this, we first analyzed the number of TICs present in the adenomas of control and SIRT6-deficient *APC*^*min*^ mice. We crossed *Sirt6*^*fl/fl*^, *VillinCre* and *APC*^*min*^ mice to obtain *Sirt6*^*fl/*+^;*VillinCre*; *APC*^*min*^ and *Sirt6*^*fl*/fl^;*VillinCre*; *APC*^*min*^ mice^3^, hereafter named *APC* and *APC; Sirt6*^*IECΔ*^, respectively. We obtained crypts from the intestines of these mice (including adenomas) and derived intestinal organoids in the absence of R-Spondin (EN medium, EGF + Noggin) (Fig. 1a). In these conditions, only adenoma stem cells (with overactivated Wnt pathway due to APC loss) can grow as organoids and, therefore, the number of organoids obtained is indicative of the stem cell activity present in the original culture^7^. Loss of SIRT6 in the intestinal epithelium of *APC*^*min*^ mice resulted in an increased number of adenoma-derived organoids (Fig. 1a, b), which were also larger in size (Fig. 1c). Similar results were obtained when we tested organoid formation from adenoma-derived single cells (Fig. 1d). In agreement with this, overexpression of SIRT6 in *APC; Sirt6*^*IECΔ*^ organoids decreased both organoid number and size, and this effect was partially dependent on SIRT6 enzymatic activity as overexpression of the catalytically dead mutant SIRT6-HY failed in impairing organoid growth and only exhibited a trend towards diminishing organoid numbers (Supplementary Fig 1a–c). Together, these data support a role for SIRT6 in regulating the number of TICs in intestinal tumors.

Based on a growing amount of data, it has been proposed that ISCs (or other epithelial cells acquiring stem cell properties under appropriate conditions) are the cell of origin of intestinal cancer^4–8^. Thus, we reasoned that an expansion of the ISC pool could explain the increased number of TICs observed in *APC; Sirt6*^*IECΔ*^ mice. Indeed, using in situ hybridization experiments we find a 25% increase in the number of cells positive for the ISC marker *Olfm4* in the intestine of *APC; Sirt6*^*IECΔ*^ mice compared to control *APC* animals (Fig. 1e and Supplementary Fig 2a). This increase in ISC number was independent of cell proliferation and changes in Wnt signaling (Supplementary Fig. 2b–d). To confirm this result and specifically address the role of SIRT6 on non-transformed ISCs, we performed intestinal organoids formation assays using control and *Sirt6*^*fl/fl*^*; VillinCre* (*Sirt6*^*IECΔ*^) mice. In this case, we cultured isolated intestinal crypts in ENR medium (EGF + Noggin+R-Spondin) to support the formation of organoids from non-transformed ISCs (Fig. 1f)^12^. As shown in Fig. 1f, we observed a significant increase in the number of organoids derived from SIRT6-deficient mice, indicating that lack of SIRT6 leads to an expansion of the ISC pool. However, the number of *Olfm4* positive cells in the intestines of *Sirt6*^*IECΔ*^ mice was not changed compared to control animals (Supplementary Fig. 2e), suggesting that SIRT6 specifically restrains ISCs expansion under unique conditions (APC loss or ex vivo plating for organoid formation), as discussed below.

Then, we sought to determine the mechanism by which SIRT6 loss drives this increase in ISCs and TICs. We focused our attention on glucose metabolism, as we previously showed that inhibition of glycolysis in vivo in *APC; Sirt6*^*IECΔ*^ mice significantly reduced the number, size, and aggressiveness of intestinal tumors^3^. We first treated mice with Dichloroacetate (DCA), an inhibitor of Pdk1. Pdk1 is a key glycolytic enzyme that phosphorylates and inhibits the activity of PDH, thus favoring aerobic glycolysis. Treatment with DCA completely rescued the increased number of *Olfm4* positive ISCs in the intestines *APC; Sirt6*^*IECΔ*^ (Fig. 1e). Similarly, organoid formation from *Sirt6*^*IECΔ*^ mice was reduced when these animals were treated with DCA, resulting in similar amount of organoids compared to control mice (Fig. 1f). To confirm these results, we knocked-down *Pdk1* in *APC; Sirt6*^*IECΔ*^ organoids and saw reduced organoid formation and growth (Supplementary Fig. 3a–c), further supporting the involvement of glucose metabolism in Sirt6-driven tumor initiation. Together, these data indicate that increased glucose metabolism upon SIRT6 loss drives ISCs expansion, which in turn translates into an increase in TICs, thus suggesting that the increased number of intestinal tumors observed in *APC; Sirt6*^*IECΔ*3^ may be defined at a very early stage during the process of transformation.

### High PDK activity defines a specific subset of ISCs expressing + 4 ISCs and EE markers

**Fig. 2:**
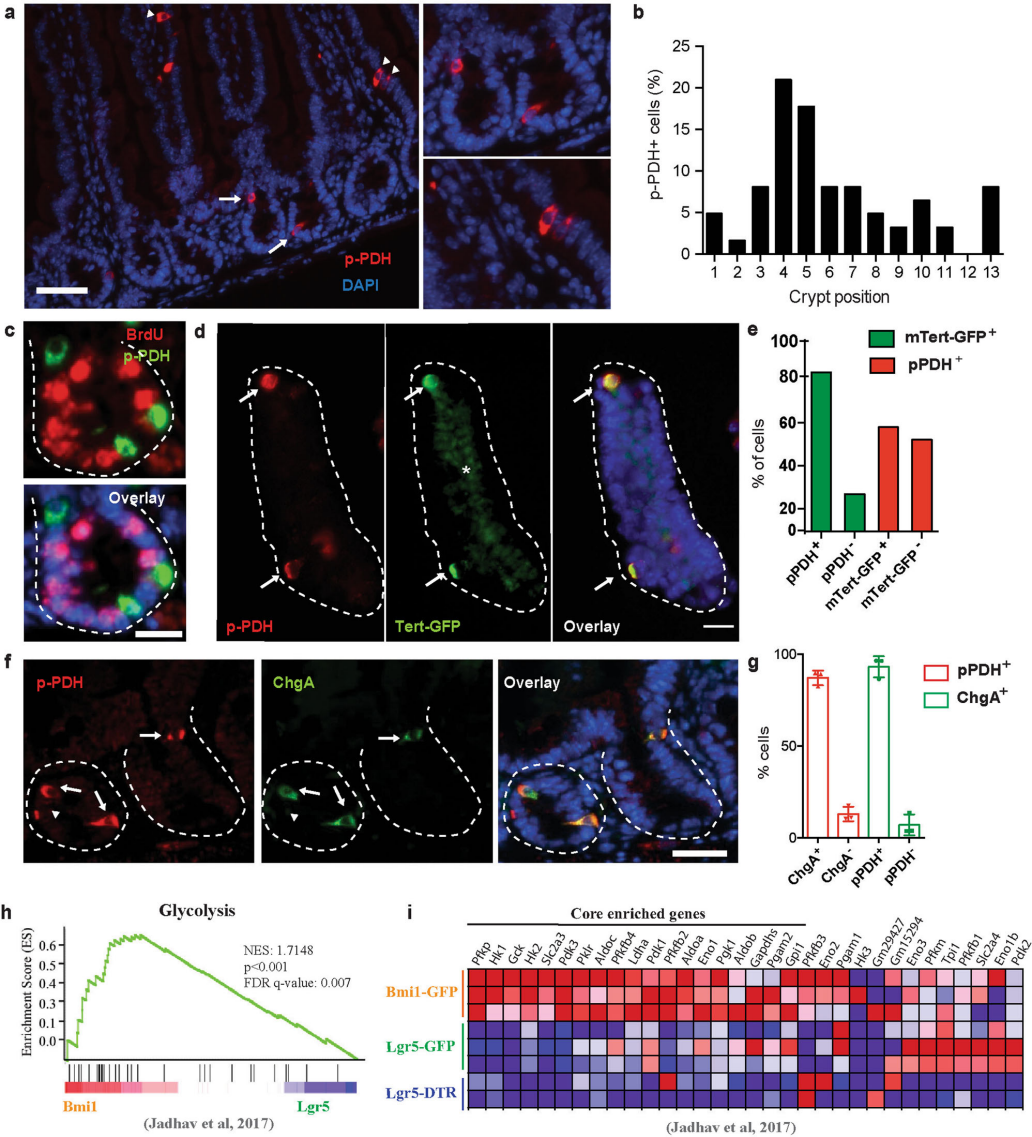
High PDK activity defines +4 ISCs from EE lineage. **a** Immunofluorescence of pPDH in small intestinal sections. pPDH^+^ cells are present in both crypts (arrows) and villi (arrowheads). On the right, magnification of intestinal crypts and villi containing pPDH^+^ cells. Scale bar, 100 μm. **b** Percentage of pPDH^+^ cells in every crypt position (62 pPDH^+^ cells form four mice were scored). **c** Double immunofluorescence for pPDH and BrdU. Representative image of four different mice is shown. Scale bar, 25 μm. **d** pPDH immunofluorescence on isolated crypts from mTert-GFP mice. Arrows mark pPDH+mTert+ cells. Asterisk marks non-specific fluorescence signal. Scale bar, 50 μm. **e** Quantification of pPDH^+^, mTert^+^, and pPDH^+^mTert^+^ cells in the intestinal crypts form three mTert-GFP mice. A total of 68 crypts were scored. **f** Double immunofluorescence for pPDH and ChgA on intestinal sections. Arrows indicate double positive cells, arrowheads indicate pPDH^+^ChgA^*−*^ cells. Scale bar, 50 μm. **g** Quantification of pPDH^+^, ChgA^+^, and pPDH^+^ChgA^+^ cells on intestinal sections from three mice (at least 50 crypts/mouse were scored). Data are presented as mean ± SEM. **h** Gene set enrichment analysis of positive regulators of glycolysis in Bmi1-GFP cells compared to Lgr5 ISCs from Lgr5-GFP and Lgr5-DTR mice (Jadhav et al.^20^). **i** Heat map of the glycolytic gene signature from (**a**) showing the core enriched genes. Source data are provided as a Source Data file.

Different from other somatic stem cells, Lgr5+ ISCs are continuously proliferating, suggesting that such glycolytic activity may represent a unique adaptation of these ISCs. Indeed, a recent report showed that glycolysis is more prominent at the base of the intestinal crypts, where ISCs reside^9^, although conflicting results have been found on whether they are ISCs themselves or other cells present in their niche the ones having high rates of glucose metabolism^10,11^. To shed further light on this issue and to identify intestinal epithelial cells with specific glycolytic features at single cell resolution, we followed the activity of PDH as a marker of glucose metabolism, since PDH catalyzes the key rate-limiting conversion of pyruvate into acetyl-CoA and its entry into the mitochondrial TCA cycle, thus controlling the switch between oxidative phosphorylation and aerobic glycolysis^13^. PDH activity is mainly regulated by PDKs, which phosphorylate and inactivate the enzyme, promoting aerobic glycolysis. Staining of intestinal sections from control mice revealed the presence of rare epithelial cells with high levels of phospho-PDH (pPDH), which were present in both crypts and villi (Fig. 2a and Supplementary Fig. 4a). Surprisingly, the number and localization of these cells were not reminiscent of Lgr5+ ISCs. Indeed, staining of intestinal sections from *Lgr5*^*eGFP−CreERT2*^ mice showed that pPDH^+^ cells were Lgr5 negative (Supplementary Fig. 4d, e). In line with this, although present in every crypt position, we found that most pPDH^+^ cells were localized in the +4/+5 position (Fig. 2b). More importantly, these cells were not proliferating, as evidenced by lack of BrdU and Ki67 staining (Fig. 2c and Supplementary Fig. 4b, c). Both their position within the crypt and their quiescent status suggested that pPDH^+^ cells could be +4 ISCs, a unique population of cells with stemness potential^14^. To confirm this possibility, we obtained crypts from mTert-GFP reporter mice, which allow the direct visualization of quiescent +4 ISCs^15^, and stained them for pPDH (Fig. 2d). We found that ~80% of *mTert*^*+*^ cells were pPDH^+^ (Fig. 2e, green bars), suggesting that +4 ISCs are highly glycolytic. On the other hand, 50% of pPDH^+^ cells were negative for *mTert* expression (Fig. 2e, red bars), suggesting the presence of some heterogeneity within pPDH^+^ cells. In line with this, other +4 ISCs markers have been described, such as Bmi1, Lrig1, and Hopx, with sometimes not overlapping expression patterns^16–18^. Recently, a common gene expression signature has been found for +4 ISCs isolated from different mouse reporters, which has identified these cells as being from EE lineage^19,20^. Therefore, we decided to stain for EE markers. Immunofluorescence co-localization revealed that 90% of pPDH^+^ cells expressed the EE marker ChgA and vice versa (Fig. 2f, g). Furthermore, electron microscopy experiments identified pPDH^+^ cells as pyramid-shaped with abundant pleomorphic secretory granules (Supplementary Fig 5a), common features of EE cells.

To confirm their glycolytic phenotype, we analyzed whether these EE cells exhibit a glycolytic signature by performing gene set enrichment analyses on a recently published RNA-seq data from Bmi1-GFP^+^ cells^20^. We found that, compared to Lgr5^+^ ISCs, Bmi1-GFP^+^ cells were significantly enriched in genes coding for positive regulators of glycolysis (Fig. 2h, i). Among the “core-enriched genes” we found enzymes of the glycolytic pathway to be upregulated (Fig. 2i), including hexokinases (*Hk1*, *Hk2*), glucose phosphate isomerase (*Gpi1*), phospho-fructokinase (*Pfkp*), aldolases (*Aldoa, Aldob*), glyceraldehyde-3-phosphate dehydrogenase (*Gapdhs*), phosphoglycerate kinase (*Pgk1*), phosphoglycerate mutase (*Pgam2*), enolase (*Eno1*) and pyruvate kinase (*Pklr*). Moreover, *Pdk1* and *Pdk3* were also among the most enriched genes, thus validating our pPDH staining. Importantly, lactate dehydrogenase A (*Ldha*) was also upregulated, which together with high PDK activity, strongly suggests that these cells are experiencing a “Warburg-like” phenotype. To validate these results, we next analyzed the expression of this glycolytic signature in a different RNA-seq dataset of stem cells from the EE lineage (including Bmi1-GFP^+^ and mTert-GFP^+^ cells)^19^. As shown in Supplementary Fig. 5d, most of these genes were also upregulated in both Bmi1-GFP^+^ and mTert-GFP^+^ cells in this dataset. Importantly, out of 19 core-enriched genes, 7 (37%) are common in the three populations (Supplementary Fig. 5e). Together, these results unveiled a metabolic heterogeneity within intestinal epithelial cells, where EE-like differentiated cells with stem cell potential exhibit specific metabolic properties characterized by an increase in glycolytic metabolism. Importantly, we find the same population of pPDH^+^/ChgA^+^ cells in human intestine (Supplementary Fig. 5b, c), indicating an evolutionary conserved role of glucose metabolism in these cells.

### pPDH^+^ cells exhibit stem cell activity

**Fig. 3:**
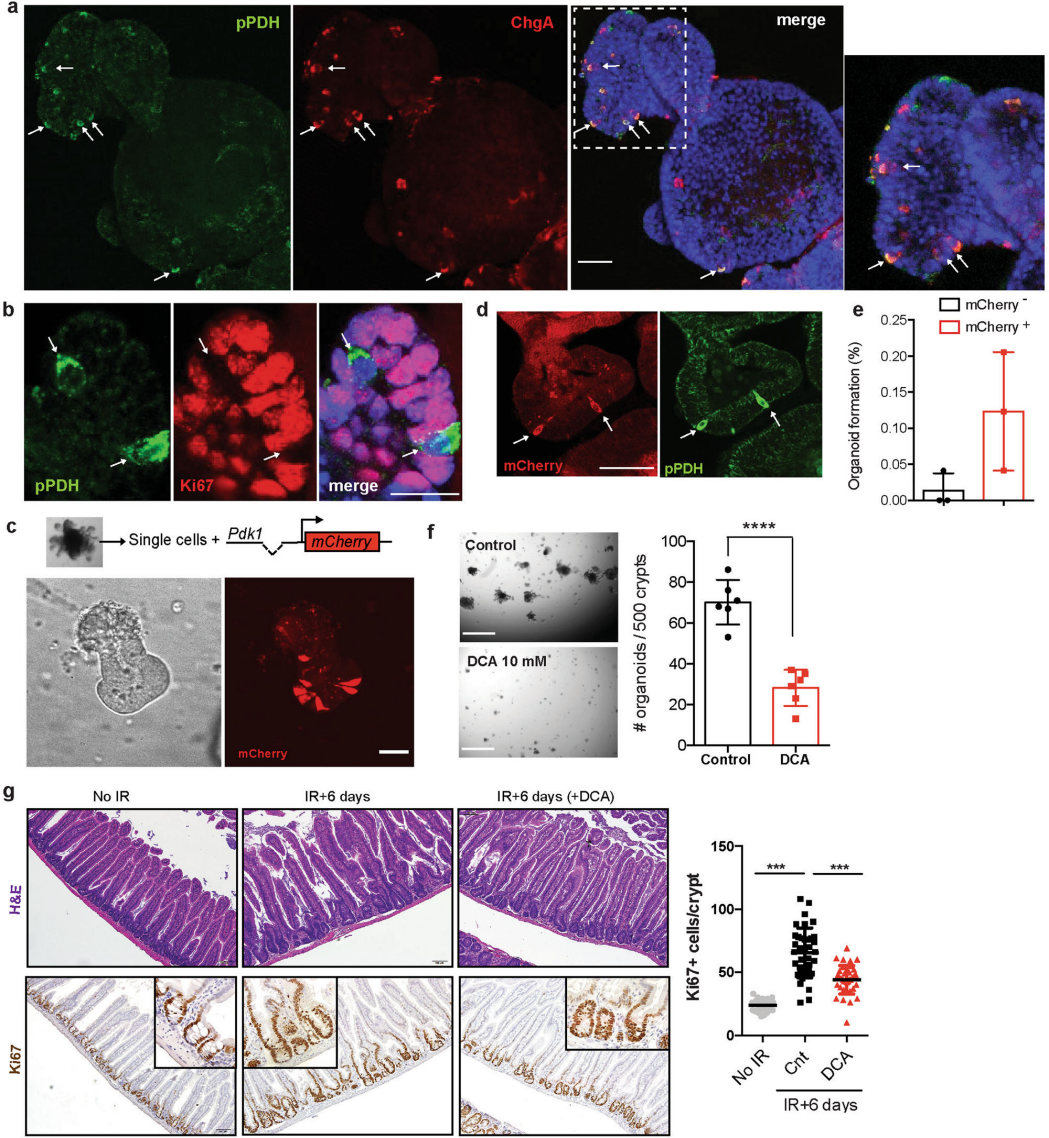
High glycolysis supports stem cell potential of pPDH^+^ intestinal epithelial cells. **a** Immunofluorescence of pPDH and ChgA in small intestinal organoids. Notice that most cells are pPDH^+^ChgA^+^ (arrows). The experiment was repeated four times with similar results. Scale bar, 25 μm. **b** Immunofluorescence of pPDH and Ki67 in intestinal organoids. The experiment was done four times with similar results. Scale bar, 20 μm. **c** Single cells derived from intestinal organoids were infected with the Pdk1-mCherry reporter. Picture shows a 3-day growing organoid with few crypt cells positive for the reporter (representative image of 10 different experiments). Scale bar, 50 μm. **d** Immunofluorescence showing co-localization of the mCherry reporter and pPDH (representative image of three independent experiments is shown). Scale bar, 50 μm. **e** FACS-sorted mCherry^*−*^ and mCherry^+^ cells were plated in matrigel and cultured in ENR medium. Organoids were counted 7 days after plating (*n* = 3 independent sorting experiments). Data are presented as mean ± SD. **f** Intestinal crypts were grown in ENR medium in the absence of presence of DCA and organoids counted at day 7 (*n* = 6 technical replicates). A representative experiment is shown. The experiment was repeated three times with similar results. Data are presented as mean ± SD. Two tail *t*-test was used to determine statistical significance between groups. Scale bar, 750 μm. **g** H&E staining (top) and Ki67 IHC (bottom) of intestinal sections of mice treated or untreated with DCA 6 days post-IR (11 Gy). The graph shows the quantification of Ki67^+^ cells in the intestines of these mice (*n* = 2 mice per condition, at least 30 crypts/mouse were scored). Two tailed *t*-test (for comparisons between two groups) or one-way ANOVA (for comparisons of more than two groups) were used to determine statistical significance between groups (*p < 0.05; **p < 0.01; ***p < 0.001). Source data are provided as a Source Data file.

To gain molecular insights into the role of glucose metabolism on these pPDH^+^ ISCs, we decided to use intestinal organoids as an ex vivo model system. First, we confirmed the presence of pPDH^+^ cells in these organoids. Indeed, we find positive cells stained with the pPDH antibody (Fig. 3a). More importantly, and similar to what we observed in the intestine, those pPDH^+^ cells were ChgA^+^ and Ki67^*−*^ (Fig. 3a, b). In order to isolate and functionally characterize these cells, we generated a reporter system where the expression of an *mCherry* cassette is driven by the mouse *Pdk1* promoter (Pdk1-mCherry). Infection of intestinal organoids with this glycolytic reporter revealed a compartmentalization of *Pdk1* expression, with only few cells expressing the mCherry reporter (Fig. 3c and Supplementary Fig. 6a). Importantly, mCherry^+^ cells stained positive for pPDH (Fig. 3d and Supplementary Fig. 6b) and were negative for Ki67 (Supplementary Fig. 6c), validating our reporter system and confirming the observed metabolic heterogeneity among intestinal epithelial cells. Next, we FACS-sorted mCherry^+^ cells and analyzed their ability to form new organoids. As shown in Fig. 3e, mCherry^+^ cells exhibited a tenfold increase in their efficiency to form organoids compared to mCherry^*−*^ cells, indicating that pPDH^+^ cells have robust stem/progenitor activity. These results are in agreement with recent findings describing EE cells as reserve ISCs^19,20^, and suggest that glucose metabolism could be actively regulating stem cell fate in these cells.

Given the increased organoid formation ability displayed by Pdk1-mCherry^+^ cells (Fig. 3e), we reasoned that inhibition of glycolysis could impair their stem cell activity. To test this, we cultured freshly isolated crypts and perform organoid formation experiments in the presence of DCA. We found that DCA treatment severely compromised the ability of intestinal crypts to form organoids (Fig. 3f), and the few that managed to grow were smaller and with very few crypt-like structures budding out of the central lumen (Supplementary Fig. 6d), indicative of poor stem cell activity. One key feature of +4 ISCs/EECs is their radioresistance and activation upon injury to repopulate the intestinal epithelium^15,17,19^. Therefore, we decided to test whether glycolysis is necessary for such intestinal regeneration. DCA treatment of mice irradiated with a single dose of 11 Gy significantly reduced the regeneration of the intestinal epithelium, as reflected by a decrease in Ki67 positive cells within intestinal crypts 6 days post-irradiation (Fig. 3g). Taken together, these results indicate that PDK activity is a functional marker of +4 ISCs/EECs and support a role for glucose metabolism in regulating stem cell activity in the intestine.

### Metabolic heterogeneity within intestinal adenomas defines cells with different stem cell activity

**Fig. 4:**
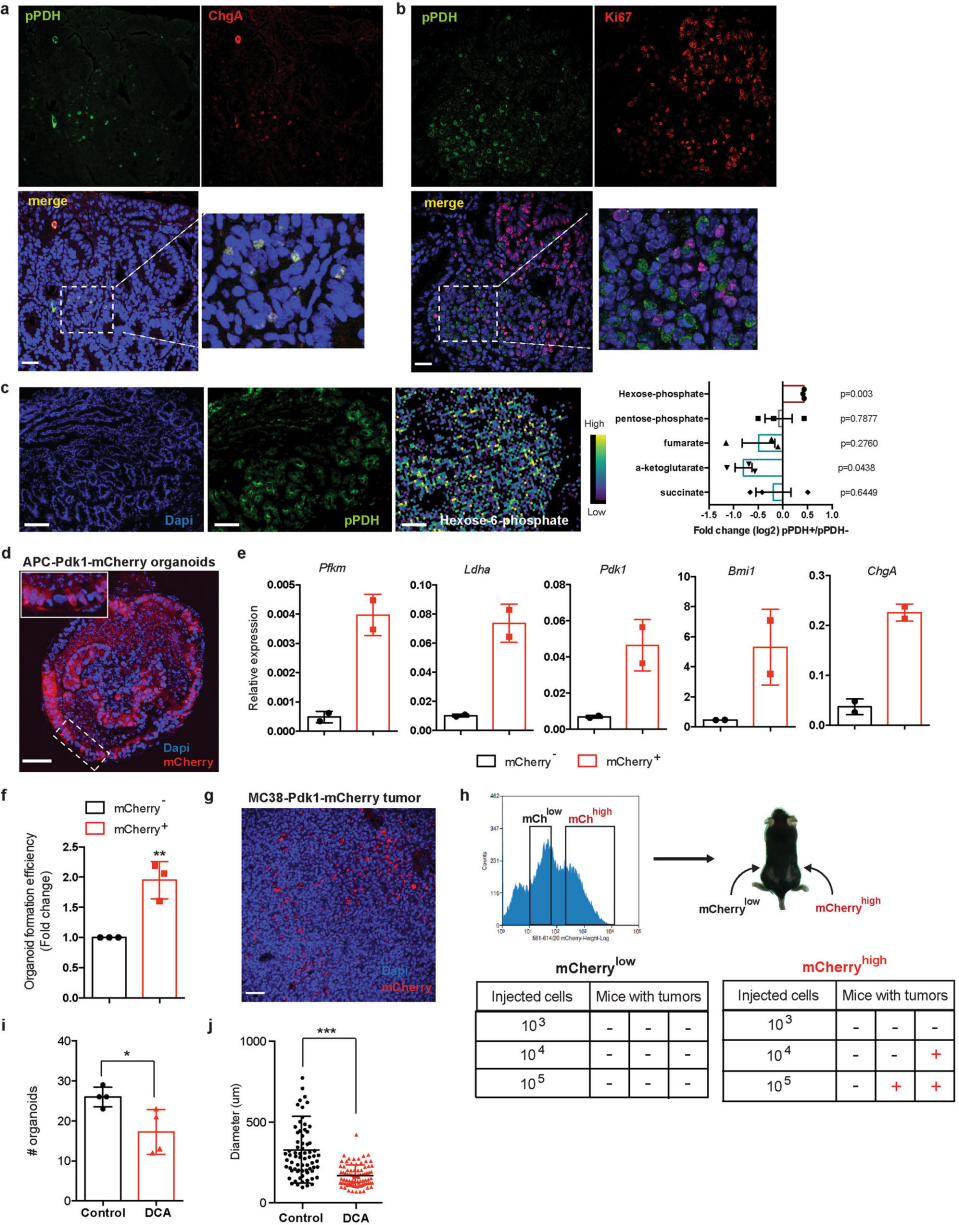
High tumor initiating potential of pPDH^+^ cells from intestinal adenomas. **a** Immunofluorescence for pPDH and ChgA of intestinal adenomas from *Apc*^min^ mice. A representative image of 24 adenomas from 6 mice is shown. Scale bar, 25 μm. **b** Immunofluorescence for pPDH and Ki67 of intestinal adenomas from *Apc*^min^ mice. A representative image of four adenomas from two mice is shown. Scale bar, 25 μm. **c** MALDI-MSI experiment showing relative abundance of hexose-phosphate in pPDH+ areas compared to pPDH− areas of an intestinal adenoma (pPDH staining of a consecutive section is shown, image is representative of three independent acquired sections from a single adenoma). Bar plot represents the fold change (average log2FC) of indicated metabolites of pPDH+ compared to pPDH− areas (one sample *t*-test *p* values are shown). *n* corresponds to a 100 μm (10 pixels of 10 μm size) of either pPDH+ or pPDH− clusters of cells. Data are presented as mean ± SEM. Scale bars, 100 µm. **d** Two photon microscopy image of a full-grown organoid from a single adenoma cell infected with the Pdk1-mCherry reporter. Organoids from three different APC^min^ mice were infected (a representative image is shown). Scale bar, 50 μm. **e** qPCR analysis showing RNA expression of indicated genes on mCherry sorted cells from two *APC*^min^-Pdk1-mCherry organoid lines (*n* = 2). Data are presented as mean ± SEM. **f** Single mCherry^low^ and mCherry^high^ cells FACS-sorted from *Apc*^min^*-*derived organoids expressing the Pdk1-mCherry reporter were plated in EN medium and organoids formed counted at day 7. The bar plot shows the quantification of three independent sorting experiments of two different clones. Error bars indicate SD. **g** mCherry expression in a subcutaneous tumor from MC38-Pdk1-mCherry cells (a representative image of six different tumors is shown). Scale bar, 50 μm. **h** mCherry^high^ and mCherry^low^ sorted cells were injected at the indicated cell dilutions in the flanks of C57BL6/J mice to analyze tumor-initiating potential. **i** Intestinal crypts from *Apc*^min^ mice were grown in EN medium in the absence or presence of 10 mM DCA and organoids counted at day 7 (*n* = 4 independent experiments). Data are presented as mean ± SEM. Two tailed *t*-test was used to determine statistical significance between groups (*p* = 0.028). **j** Quantification of the size of the organoids from (**i**). Error bars represent SD (*p* < 0.0001). Two tailed *t*-test was used to determine statistical significance between groups (*p < 0.05; **p < 0.01; ***p < 0.001). Source data are provided as a Source Data file.

Most epithelial tumors exhibit a similar hierarchical structure to the corresponding normal tissue from where they originated. Following this idea, we next investigated whether high levels of glucose metabolism defined a subset of cells with stem cell activity in the adenomas of *APC*^min^ mice. Similar to what we found in normal intestinal epithelium, analysis of *APC*^min^ adenomas revealed the presence of rare pPDH^+^ tumor cells (Fig. 4a, b). Notably, we also found variability in the amount of these cells among adenomas, with some adenomas having very few or even none (Supplementary Fig 7a). These results suggest the presence of intra-tumor and inter-tumor heterogeneity of glucose metabolism reprogramming within intestinal adenomas. Immunofluorescence experiments showed that pPDH^+^ tumor cells were also ChgA^+^ and Ki67^*−*^ (Fig. 4a, b), indicating that these transformed cells most likely derived from pPDH^+^ cells originally present in the normal intestine. In line with this, Lgr5+ cells in the adenomas were pPDH^*−*^ (Supplementary Fig. 7b), consistent with what we observed in normal epithelium (Supplementary Fig. 4d,e).

To map the metabolic profiling of these cells we performed matrix-assisted laser desorption ionization mass spectrometry imaging (MALDI-MSI) on frozen sections from intestinal adenomas. We co-registered both MALDI-MSI and pPDH immunofluorescence images from sequential sections, which allowed us to appreciate the relative abundance of specific metabolites in different tumor subpopulations. Although the current MALDI-MSI technology does not achieve single-cell resolution, we were able to obtain 10 μm spatial resolution, which enabled us to determine the relative abundance of metabolites in clusters of pPDH+/pPDH− cells (Fig. 4c). Our results showed an accumulation of hexose-phosphate and a decrease in several TCA cycle intermediates (with clear statistical significance for α-KG, and a trend for succinate and fumarate) in pPDH+ cells (Fig. 4c and Supplementary Fig. 7c), indicating that these cells exhibit active glycolysis and reduced mitochondrial TCA cycle. Despite having increased glycolytic activity, the levels of ribose-5-phosphate were not changed in pPDH+ cells compared to pPDH− cells (Fig. 4c), a result that is consistent with these cells being quiescent and, thus, not having an increased requirement to build up nucleotides from ribose-5-phosphate.

To confirm these results and to obtain an in-depth analysis of these cells at single cell resolution, we turned again to an in vitro system and derived intestinal organoids from *APC*^min^ tumors (as described above). We first confirmed the presence of these highly glycolytic quiescent EE-like cells in *APC*^min^ organoids, which exhibited a similar pattern to what was observed in *APC*^min^ adenomas (Supplementary Fig. 7d). Then, we transduced these organoids with the *Pdk1-mCherry* reporter (Fig. 4d) and FACS-sorted cells derived from these organoids according to *mCherry* expression. qPCR analysis showed high levels of *ChgA*, *Bmi1*, and glycolytic gene expression in Pdk1-mCherry^+^ cells compared to Pdk1-mCherry^*−*^ (Fig. 4e), indicating that pPDH+ cancer cells exhibit the same cell phenotype than pPDH+ cells in the normal epithelium. Importantly, these results support our MALDI-MSI data and indicate that pPDH+ cells are highly glycolytic. Consistent with the results observed in untransformed pPDH^+^ cells, sorted Pdk1-mCherry^+^ tumor cells displayed an increased ability to form new organoids (Fig. 4f), suggesting that this cell population is enriched in TICs. Remarkably, injection of Pdk1-mCherry organoids into the flanks of immunodeficient mice formed tumors with mCherry^+^ cells that were negative for the proliferation marker Ki67 (Supplementary Fig. 7e), indicating that the metabolic heterogeneity observed in intestinal adenomas can be recapitulated in these organoid-derived tumors, thus validating this ex vivo system for further analyses. To demonstrate the tumor initiating potential of these cells, we took advantage of the mouse colon cancer cell line MC38, which we transduced with the Pdk1-mCherry reporter to generate MC38-Pdk1-mCherry cells. Injection of these cells into the flanks of syngeneic C57BL6/J mice gave rise to tumors that exhibited an heterogenous pattern of mCherry expressing cells (Fig. 4g), confirming the metabolic heterogeneity observed in *APC*^min^ adenomas. MC38-Pdk1-mCherry tumors were digested and mCherry sorted cells were injected back into C57BL6/J mice (Fig. 4h). While no tumors were formed by injection of Pdk1-mCherry^low^ cells, even at the higher concentration, 1 out of 3 (when 10^4^ cells were injected) and 2 out of 3 (when 10^5^ cells were injected) mice injected with Pdk1-mCherry^high^ cells developed tumors (Fig. 4h). This result demonstrates that Pdk1-mCherry+ cells are endowed with tumor initiating potential. Finally, to test if active aerobic glycolysis is functionally relevant for the stem cell activity of pPDH^+^ tumor cells, we performed organoid formation experiments in the presence of DCA. As observed for non-transformed organoids, we found that DCA treatment severely impaired both number and size of *APC*^min^ organoids (Fig. 4i, j). Together, these results are in agreement with glucose metabolism regulating stem cell activity in the intestine and establish a metabolic hierarchy in intestinal adenomas, where highly glycolytic, quiescent cells retain tumor initiating potential.

### High glycolytic activity protects pPDH^+^ stem cells from oxidative stress

**Fig. 5:**
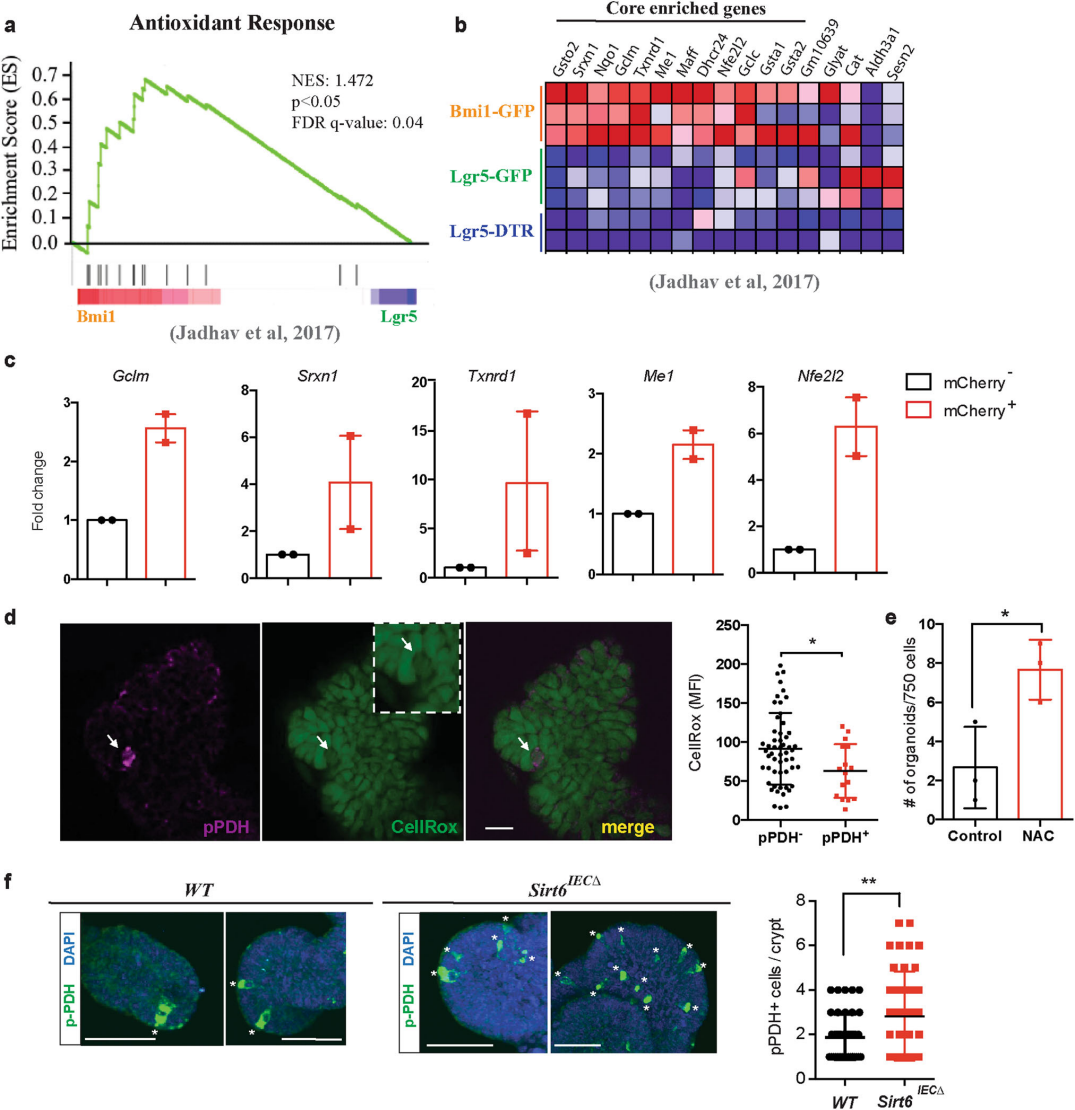
Increased antioxidant response in pPDH^+^ cells. **a** Gene set enrichment analysis of antioxidant genes in Bmi1-GFP cells compared to Lgr5 ISCs from Lgr5-GFP and Lgr5-DTR mice (Jadhav et al.^20^) (nominal *p* value = 0.013). **b** Heat map of the antioxidant gene signature from (a) showing the core enriched genes. **c** qPCR analysis showing RNA expression of indicated genes on mCherry sorted cells from two *APC*^min^-Pdk1-mCherry organoid lines (*n* = 2). Data are presented as mean ± SEM. **d** Data represent CellRox-Green MFI of 16 pPDH+ cells from ten organoids. For each pPDH+ cell, 5–6 adjacent pPDH− cells were analyzed (total of 54 pPDH− cells from 10 organoids). Data are presented as mean ± SD (*p* = 0.0257). Scale bars, 25 µm. **e** Organoid formation efficiency of Apc; Sirt6 ^Δ^IEC organoids treated with 200 μM NAC. Data shown are from one experiment performed in triplicate, representative of *n* = 2. Data are presented as mean ± SD (*p* = 0.0285). **f** Immunofluorescence of pPDH in intestinal organoids from control and *Sirt6*^*IECΔ*^ mice (representative crypts are shown). Dot plot shows the quantification of pPDH^+^ cells in a total of 40 crypts from organoids derived from two control and two *Sirt6*^*IECΔ*^ mice (*p* = 0.0073). Error bars indicate SD. Scale bars, 20 μm. Two tailed *t*-test was used to determine statistical significance between groups (*p < 0.05; **p < 0.01; ***p < 0.001). Source data are provided as a Source Data file.

The results above indicate that glucose metabolism in pPDH^+^ cells is not rewired to support cell proliferation or biomass production. A switch towards glycolysis also provides cells with increased reduced glutathione and NADPH, an important adaptation against oxidative stress^21^. In addition, previous work in long-lived stem cells, particularly in the hematopoietic system, demonstrated increased antioxidant features, an important adaptation to avoid chronic exposure to DNA damage^22^. Therefore, we decided to test whether these pPDH^+^ cells may use this switch to protect themselves against oxidative stress. Analysis of the RNA-seq data from Bmi1-GFP^+^ cells^20^ showed a significant enrichment of “Antioxidant response” genes, compared to Lgr5-GFP^+^ ISCs (Fig. 5a). Among the core-enriched genes, we found antioxidant enzymes (*Srxn1*, *Txnrd1*, *Dhcr24*), glutathione metabolism genes (*Gsto1*, *Gsta1*, *Gclm*, *Gclc*), NADPH metabolism genes (*Nqo1*, *Me1*) and key transcription factors from the bZIP family with known roles in regulating the expression of antioxidant genes (*Maff*, *Nfe2l2*) (Fig. 5b). Importantly, as observed for glycolytic genes, the expression of most of these genes was also upregulated in both Bmi1-GFP^+^ and mTert-GFP^+^ cells from the RNA-seq dataset of Yan and colleagues^19^ (Supplementary Fig. 8a,b), indicating that stem cells from EE origin exhibit a robust antioxidant signature. To directly validate this analysis, we sorted Pdk1-mCherry^+^ cells from *APC; Sirt6*^Δ*IEC*^ organoids and assessed the expression of these genes by RT-PCR. Indeed, Pdk1-mCherry+ cells exhibited higher expression levels of the antioxidant genes *Gclm*, *Srxn1*, *Txrnd1*, *Me1*, and *Nrf2* compared to Pdk1-mCherry^*−*^ cells (Fig. 5c). Furthermore, our MALDI-MSI experiment on frozen adenomas revealed an enrichment of oleic and palmitoleic acids in pPDH+ cells compared to pPDH− cells (Supplementary Fig. 8c). Accumulation of these monounsaturated fatty acids has been associated with protection against oxidative stress and, in particular, with a decrease in membrane lipid peroxidation^23^, thus linking high glycolysis and antioxidant activity in pPDH+ cells.

To directly determine whether pPDH^+^ cells exhibited decreased levels of ROS, we stained intestinal organoids with the specific ROS dye CellRox and observed that pPDH^+^ cells accumulated 30% less dye (Fig. 5d and Supplementary Fig. 8d), indicating decreased levels of ROS in these cells. Next, we assessed the functional role of ROS on stem cell potential. We performed organoid formation experiments with *APC; Sirt6*^Δ*IEC*^ shPDK1 organoids, which showed decreased stem cell potential (Supplementary Fig. 3a–c), in the presence of the antioxidant N-acetyl-cysteine (NAC). If ROS generation due to impaired glycolytic metabolism would account for this decreased stem cell potential, we would expect this phenotype to be rescued by treatment with NAC. Indeed, as shown in Fig. 5e, we observed a two-fold increase in organoid formation potential in the presence of NAC. Together, these results support a model in which active glycolysis promotes stem cell potential of pPDH^+^ cells by keeping ROS levels low and are consistent with the increased organoid formation in the absence of SIRT6 (Fig. 1a, f), where glucose metabolism is further augmented. Indeed, analysis of organoids from WT and *Sirt6*^*IECΔ*^ mice revealed an expansion of pPDH^+^ cells in the absence of SIRT6 (Fig. 5f), providing an explanation for the increased tumorigenesis upon SIRT6 loss.

## Discussion

Our previous findings indicated that SIRT6-dependent metabolism could drive tumorigenesis independently of activation of canonical signaling pathways^3^. In this study, we took advantage of a model of APC-driven intestinal tumorigenesis to demonstrate that glucose metabolism promotes tumor initiation by controlling stem cell activity and TICs in the intestine. Of note, fatty acids and phospholipid metabolism have been also involved in intestinal tumorigenesis by regulating the number and activity of Lgr5+ cells^24,25^, highlighting the relevance of the metabolic regulation of stem cell fate on cancer initiation and growth, and that different cells with stemness potential rely on distinct metabolic pathways. Interestingly, calorie restriction enhances the number of Lgr5^+^ ISCs by increasing the activity of SIRT1^26^, suggesting that sirtuins could have evolved as critical mediators coupling changes in metabolism with ISC fate.

Several recent studies have provided evidence linking glucose metabolism to ISC function. It has been shown that Lgr5^+^ ISCs exhibit a very low rate of mitochondrial pyruvate oxidation due to low levels of the mitochondrial pyruvate carrier in these cells^11^. Based on this observation, it was speculated that Lgr5^+^ ISCs could rely on glycolysis for their function, although no direct proof was provided. Moreover, despite the low levels of pyruvate oxidation, mitochondrial metabolism was not abated in these cells, indicating that mitochondria in Lgr5^+^ ISCs could be involved in other metabolic programs, including oxidation of fatty acids or other carbon sources. Indeed, another recent report demonstrated that Lgr5^+^ ISCs have an increased oxidative metabolism, which is supported by the uptake of lactate secreted by the more glycolytic Paneth cells^10^. In agreement with these observations, we did not find Lgr5^+^ ISCs to be highly glycolytic, even though their expansion upon SIRT6 loss was dependent on glucose metabolism. Instead, by employing an unbiased approach we uncovered a rare population of IECs exhibiting high PDK activity, which we identified with similarities to the recently described +4 cells from EE origin^19,20^. Although we could not perform metabolomic experiments on this rare population of IECs at single cell resolution, our gene expression analysis and our MALDI-MSI experiments suggest that pPDH+ cells are highly glycolytic. We cannot rule out that other IECs could be highly glycolytic, yet the position of pPDH^+^ cells, their quiescent status, the high overlapping of pPDH and ChgA staining, and the glycolytic signature expressed by Bmi1-GFP^+^ and mTert-GFP^+^ cells indicate that these cells are +4 cells from EE origin. In line with this, we find only a minor fraction (3%) of Paneth cells being glycolytic based on pPDH staining (Supplementary Fig 4f). Importantly, high glycolysis in these EE-like cells is functionally relevant, as its inhibition severely impairs stem cell activity and tumorigenic potential both in vitro and in vivo. A caveat of our DCA experiments is that all epithelial cells are exposed to this glycolytic inhibitor. However, due to the high PDK activity showed by pPDH^+^ cells compared to any other cell type in the intestine, it seems conceivable to reason that the biological effects of DCA (a PDK inhibitor) could be explained by specifically targeting these cells. In line with this, Lgr5+ ISCs mostly rely on mitochondrial oxidation (as stated above) and, therefore, it seems unlikely that inhibiting glycolysis in these cells could account for the impaired organoid formation in the presence of DCA. Moreover, our experiments showed that a very small fraction of glycolytic Paneth cells are pPDH^+^, suggesting that, if any, the effect of DCA on Paneth cell fate would be very limited.

An important result of our study is that rather than being used to support biomass production and proliferation, our data suggest that high glycolysis in pPDH^+^ cells is diverted towards the generation of reduced power to keep ROS levels low. This is a well-known mechanism to maintain stemness and avoid cell differentiation of hematopoietic stem cells^27^, which, indeed, it is regulated by PDK activity^28^. In agreement with this, we found lower levels of ROS in Pdk1-mCherry^+^ cells, suggesting a conserved mechanism to preserve stem cell potential among adult quiescent stem cells.

Finally, similar to the normal intestine, we found that these pPDH^+^ cells are also present in intestinal adenomas and exhibit the same features as non-transformed pPDH^+^ cells. Importantly, our MALDI-MSI experiments showed that tumor areas enriched in pPDH+ cancer cells exhibited an accumulation of glycolytic intermediates and a downregulation of TCA cycle metabolites, suggesting that pPDH+ cancer cells have active glycolysis and decreased mitochondrial activity (consistent with high PDK activity and inhibition of pyruvate oxidation). However, a major limitation of this technique is that because of its resolution of ~10 μm, it does not allow to obtain the metabolic profiling of single cells. In this context, our genetically-encoded metabolic reporter (Pdk1-mCherry) enabled us to visualize, sort and analyze cells with different metabolic activity at single cell level. A limitation of our reporter is that performing metabolomic studies of sorted rare cell populations is extremely challenging and, thus, our studies are based on gene expression analyses. Nevertheless, the results obtained by MALDI-MSI and Pdk1-mCherry reporter-based experiments combined are in agreement with pPDH+ cells being a highly glycolytic quiescent cell population exhibiting a strong antioxidant response. Although it is not clear at the moment what is the precise contribution of these cells to tumor formation, our results support a model where these quiescent highly glycolytic cells could give rise to Lgr5^+^ ISCs under certain stress conditions (such as APC mutation and loss), which in turn would be responsible for tumor initiation. In agreement with this, we observe increased number of both pPDH^+^ cells and crypt base columnar (CBC) ISCs (positive for Olfm4) upon SIRT6 loss, which translates into enhanced tumor initiating potential. Importantly, this increase in CBC ISCs is not due to an increase in cell proliferation, but rather it suggests a dedifferentiation process from pPDH^+^ cells to CBC ISCs. However, another scenario in which pPDH^+^ cells could be cell of origin of intestinal tumors may be possible, as it has been shown that activation of β-catenin in EE progenitors leads to the formation of adenomas^29^. Further genetic and lineage-tracing experiments will be required to test these hypotheses.

Taken together, our results define SIRT6-dependent glucose metabolism reprogramming as an early event during tumorigenesis, promoting tumor initiation by increasing the number of cells that can give rise to a tumor. By tracking down the origin of these tumor-initiating cells, we have uncovered a highly glycolytic quiescent population of cells with EE features and strong stem cell potential. High glycolytic activity protects these cells from ROS accumulation and supports stem cell activity, in turn driving tumor formation. More importantly, we found that even within tumors, glycolysis is present in only a fraction of cells, and these are as well non-proliferative. These results indicate that, at least in this particular case, a “Warburg-like” adaptation evolved to enhance reducing power rather than to support biomass. Furthermore, the study indicate that metabolism contributes to cancer heterogeneity, similar to what it has been proposed for genetic and epigenetic events. Overall, our results highlight a driving role for glucose metabolism in the control of stem cell fate and tumorigenesis and identify TICs as a cell of origin for the Warburg effect.

## Methods

### Mice

All mice were maintained in compliance with the guidelines of the Institutional Animal Care and Use Committee (IACUC) of the Massachusetts General Hospital (approval#2019N000111), the Ethical Commission of the Candiolo Cancer Institute and the Italian Ministry of Health (approval# 185/2018-PR). We used *Sirt6*^*fl/fl*3^, C57BL/6J-*Apc*^*Min*^/J mice (Jackson Laboratories), C57BL6/J *Villin 1-Cre* (gift from K. Haigis) and *Lgr5*^*eGFP−IRES−CreERT2*^ (Jackson Laboratories). Males and females (older than 6 weeks) were used for experiments. In vivo DCA treatment was performed as previously described^3^. For intestinal injury, mice were exposed to 11 Gy of whole body irradiation and tissue was collected at indicated time points. DCA-treated group was provided with DCA (Sigma, 347796)) in the drinking water (5 g/l) 16 h before irradiation and kept on treatment after the end of the experiment. Subcutaneous injection of organoids was performed as previously described^30^. Briefly, organoids corresponding to 5×10^5^ cells were mixed with 100 μl of Matrigel (growth factor reduced, BD Biosciences #354230) and injected into the flanks of SCID mice (Taconics Farms, Inc., Hudson, NY). MC38 cells were injected subcutaneously into the flanks of C57BL6/J mice (Charles River Laboratories). Maximal tumor size allowed (2 cm) was not exceeded.

### Organoid culture

Small intestinal organoids were derived from isolated crypts collected from the whole small intestine of WT and *Sirt6*^*IECΔ*^ mice following the protocol by Sato and colleagues^12^. 500 crypts were plated in a 50 μl drop of Matrigel in 24-well plates and overlaid with 500 μl of ENR medium, containing basal crypt media (Advanced DMEM/F12, penicillin/streptomycin, 10 mM HEPES, 2 mM Glutamine) supplemented with 1× B27 (Gibco, 17504-044), 1× N2 (Gibco, 17502-048), 50 ng/ml rmEGF (Gibco, PMG8043), 100 ng/ml Noggin (Peprotech, 250-38) and R-Spondin1-CM (20% v/v). Crypts from *APC* and *APC; Sirt6*^*IECΔ*^ mice were plated as above but overlaid with EN medium (ENR medium with no R-Spondin1). For single adenoma cell organoid formation, adenomas from *APC* and *APC; Sirt6*^*IECΔ*^ mice were harvested, minced and digested with 1 mg/ml collagenase type XI (Sigma, C7657) and 1 U/μl DNAse (Roche, 4716728001) for 20’ at 37 °C. Cells were filtered through a 40 μm mesh, spun down and incubated with PBS + 2 mM EDTA for 20’ at 4 °C. Cells were resuspended in basal crypt medium and seeded in 50 μl Matrigel drops (1000 cells/well) in 24-well plates. After solidification, Matrigel drops were overlaid with EN medium supplemented with Rock inhibitor Y-27632 20 ng/ml (SellectChem, 51049). Media was changed every 3 days and organoids split every 5–7 days. For organoid formation experiments, organoids were counted at day 7 and size was calculated by averaging 2 diameter measures using ImageJ software. For organoid formation experiments from FACS-sorted cells, Matrigel drops were overlaid with either ENR (supplemented with 10 μM Y-27632 and 1 μM Jagged-1) or ER medium supplemented with 10 μM Y-27632.

### In situ hybridization, immunohistochemistry, and immunofluorescence

ISH for Olfm4 was performed on formalin-fixed intestine at the specialized histopathology service core at the Brigham and Women’s Hospital using RNAscope, according to the manufacturer’s instructions. Immunohistochemistry was performed as previously described^31^. Primary antibodies were diluted in blocking solution as follows: anti-Ki67 (Cell Signaling, #9449) 1:200, anti-phospho-PDH-E1a-Ser293 (Abcam ab92696) 1:500. Stained slides were photographed with an Olympus DP72 microscope. For immunofluorescence, rehydrated slides were washed and subjected to antigen retrieval in citrate buffer (for phospho-PDH, BrdU, Ki67, and ChgA) or Tris-EDTA pH 9.0 buffer (for Lgr5-GFP) at 95 °C for 20’. Slides were let to cool down, washed with dH_2_O, PBS and TNT buffer (100 mM Tris-HCl pH 7.5, 150 mM NaCl, 0.05 % Tween-20) and blocked with TSA blocking reagent (Perkin Elmer FP1020) for 1 h at room temperature. After washing 3 times with TNT buffer, slides were incubated with the following primary antibodies in blocking reagent overnight at 4 °C: anti-Ki67 (1:200), anti-phospho-PDH-E1a-Ser293 (1:500), anti-ChgA (Santa Cruz Biotechnolgies sc-393941) 1:200, anti-GFP (Clontech 632381) 1:100. Slides were washed with TNT buffer, incubated with goat anti-rabbit Alexa Fluor-488 (Immunological Sciences, IS-20019), goat anti-mouse Alexa Fluor-555 (Immunological Sciences IS-20231) and goat anti-mouse Alexa Fluor-647 (Immunological Sciences, IS-20040)(1:500 dilution) in blocking buffer for 1 h at room temperature and counterstained with DAPI. Slides were then mounted with Fluoromount and imaged by a Leica SPE confocal microscope. For BrdU experiments, mice were i.p. injected 2 h before euthanasia with BrdU and intestines processed as above using anti-BrdU (Dako #M0744) 1:200 as a primary antibody.

### Organoid imaging

Organoids were collected with cold PBS and transferred to a 15-ml tube. They were spun down at 300 g for 3’ and fixed with 4% PFA for 1 h at room temperature. After washing twice with PBS, organoids were permeabilized with PBS-1% Triton-X-100 for 20’ at 4 °C and incubated in blocking solution (PBS + 1% BSA + 3% goat serum + 0.2% Triton-X-100) for 1 h at 4 °C. Organoids were then incubated with primary antibodies in working solution (PBS + 0.1% BSA + 0.3% goat serum + 0.2% Triton-X-100) over night at 4 °C. The primary antibodies were removed, and the samples were washed three times for 5 min each with PBS before incubating them with secondary antibodies (1:500) in working solution for 1 h at room temperature. Organoids were stained with DAPI, resuspended with mounting medium and mounted on slides. Images were acquired using a Leica SPE confocal microscope, a Leica SP8 confocal microscope or an in house-built Olympus two-photon microscope (Multiphoton Microcopy Core, Massachusetts General Hospital), depending on the experiment. For live imaging of Pdk1-mCherry, organoids were plated in Matrigel drops in 6-cm plates and imaged by two-photon microscopy using a water immersion 25× objective. To measure ROS levels in organoids, we incubated them with CellRox Green (Life Technologies, C10444) for 1 h, fixed them with 2% PFA and imaged them using a Leica SPE confocal microscope.

### Constructs and viral infection

To generate the Pdk1-mCherry reporter, we amplified around 1.3 kb upstream of the ATG of the *Pdk1* gene using genomic DNA form mouse ES cells. We cloned this promoter region into the pHAGE2-EF1aL-mCherry-W vector (generous gift from Dr. Darrell Kotton) by exchanging the EF1a promoter by the Pdk1 promoter. The 7×TCF-luciferase plasmid was a kind gift of Prof. Konrad Hochedlinger. To knock-down the expression of *Pdk1* we used the pLKO.1-shPDK1 lentiviral vector^3^. SIRT6 overexpression in intestinal organoids was done by employing the pLVX-Tet-ON/pRetro-X-TIGHT-SIRT6 system as previously described^32^. Viral particles containing the above-mentioned plasmids were synthesized using lentiviral (pCMV-dR8.91) packaging plasmid with pCMV-VSV-G (Addgene) and concentrated by ultracentrifugation. For organoid infection, we adapted the protocol from Onuma et al.^30^. Briefly, we collected organoids from 6 wells of a 24-well plate and dissociated them to single cells by Accutase treatment (10’ at 37 °C). 1–5 × 10^5^ cells single cells were resuspended in 500 μl of ENR medium (supplemented with 1 μM Jagged-1, 10 μM Y-27632 and 3 μM CHIR99021) containing 10 μg/ml polybrene and 10 μl of the concentrated virus. Cells were then seeded on a pre-solidified 50 μl drop of Matrigel in a 24-well plate and placed in the incubator overnight. Next day, medium and floating cells were removed and attached cells were covered by a second drop of 50 μl of Matrigel and overlaid with ENR medium supplemented with 1 μM Jagged-1, 10 μM Y-27632 and 3 μM CHIR99021 (Tocris, 4423). Infection of *APC*^*min*^ organoids was performed in the same way but using EN medium (supplemented with 10 μM Y-27632). For sorting experiments, single infected organoids were picked and expanded to ensure that the levels of mCherry expression corresponded to the activity of the Pdk1 promoter and not to differential efficiency of infection.

### RNA extraction and Real-time PCR

Total RNA from organoids was extracted with the TriPure Isolation Reagent (Roche) as described by the manufacturer. Total RNA from sorted cells was extracted with the NucleoSpin RNA Plus XS kit (Maherey-Nagel) following the manufacturer instructions. cDNA synthesis and Real-Time PCR were performed as previously described^3^. Briefly, total RNA was retro-transcribed by using the QuantiTect Reverse Transcription Kit (Quiagen). Real-time PCR was performed using the SYBR green master mix (Roche), following the manufacturer’s instructions, with the exception that the final volume was 12.5 μl of SYBR green reaction mix. Real-time monitoring of PCR amplification was performed using the LightCycler 480 detection system (Roche). Data were expressed as relative mRNA levels normalized to the β-actin expression level in each sample. The primer sequences are provided in Supplementary Data 2.

### Protein extraction and Western blot

Organoids were harvested and Matrigel removed by incubating them 1 h on ice with Cell Recovery Solution (Corning, 354253). Protein extracts and Western Blot were performed as previously described^3^. The antibodies used are as follows: anti-SIRT6 (Abcam, ab62739) 1:1000, anti-PDK1 (Cell Signaling, 3820) 1:1000, anti-H3K9ac (AbCam, ab12179) 1:1000 and anti-β-Actin (AbCam, ab8226) 1:5000.

### Wnt reporter assay

Wnt transcriptional activity was determined by luciferase experiments as previously described^33^. We used a puromycin-selectable lentiviral vector expressing Firefly luciferase under the control of seven multimerized Tcf binding sites (7×TCF-luciferase) together with a lentiviral vector constitutively expressing Renilla luciferase. For organoids, two *APC* lines and three *APC; Sirt6*^*IECΔ*^ lines were infected with Firefly and Renilla luciferase lentiviral vectors and selected for 7 days with 1 μg/mL puromycin. Following selection, organoids corresponding to each genotype were expanded and grown in EN meida. Luciferase activity was determined using the Dual-Luciferase Reporter Assay system (Promega, Madison, WI). For 293T cells, stably infected cells expressing 7×TCF-luciferase and Renilla were transfected with 1 μg of pCMV-3xFlag-SIRT6 (or empty vector) and luciferase activity was measured 48 h after transfection.

### RNA-seq analysis

We used the following publicly available gene expression datasets: GSE99815, GSE83394. Each fastq file (R1) was downloaded and realigned using STAR^34^. We used version mm10 of the genome and Gencode vM9 as reference. Transcriptome database and gene quantification was performed with HTSEQ^35^. Features with less than 1 read were removed from further analysis and, when multiple symbols were assigned to multiple ENSEMB genes, we selected the entry with highest mean expression. GSEA enrichment analysis was run comparing Bmi1^+^ cells vs. Lgr5^+^ cells (for GSE83394), or the ratio between either Bmi1^+^ vs. Bmi1^*−*^ or mTert^+^ vs. mTert^*−*^ compared to Lgr5^+^ vs. Lgr5^*−*^ (for GSE99815) using 1000 genesets permutations. Only genesets with FDR < 0.05 were considered^36^. As reference, we employed a list of glycolytic and antioxidant genes genesets manually curated (Supplementary Data 1) and annotated to *mus musculus* gene symbols, according to Ensembl.

### Electron microscopy and immunogold

Electron microscopy was performed in the Microscopy Core of the Center for Systems Biology/Program in Membrane Biology, Massachusetts General Hospital. Small pieces of intestine were cut in half (longitudinally) and residual undigested material removed by rinsing tissue in 4% paraformaldehyde/75 mM lysine-HCL/10 mM sodium periodate (PLP) in 0.15 M sucrose in 37.5 mM sodium phosphate^37,38^. Tissue was allowed to remain in fixative overnight @ 4 °C, then cut into smaller pieces (roughly 1 mm × 1 mm) with a specific orientation to allow for landmarking edges during embedding. Specimens were rinsed several times in PBS and dehydrated through a graded series of ethanol solutions to 100%. Tissue pieces were then infiltrated with 100% LR White resin (EMS, Hatfield, PA) for a minimum of two hours at room temperature on a rotator, then transferred into fresh LR White resin in gelatin capsules and allowed to polymerize 24–48 h at 50 °C.

Ultrathin (70 nm) sections were cut using a Reichert Ultracut E ultramicrotome and collected onto formvar-coated grids (EMS, Hatfield, PA). Grids were incubated for one hour at room temperature on drops of primary antibody (rabbit polyclonal antiPDH-E1a-Ser293, Abcam #92696) diluted 1:100 in DAKO antibody diluent, then rinsed several times in PBS. Specimens were incubated on drops of a secondary gold conjugate for one hour at room temperature (goat anti-rabbit IgG, 15 nm, Ted Pella #15727). Grids were rinsed several times with distilled deionized water and contrast-stained using 2.0% aqueous uranyl acetate. Grids were examined at 80 kV in a JEOL 1011-transmission electron microscope (Peabody, MA) equipped with an AMT digital camera and proprietary image capture software (Advanced Microscopy Techniques, Danvers, MA).

### Tissue preparation for MALDI MSI and immunofluorescence microscopy

Colorectal tissue was stored at −80 °C until processing. Cryosections of the colorectal tissue, containing the adenoma, were taken at 10 µm thickness and were mounted on indium tin oxide slides for MALDI MSI analysis. Serial sections were obtained for MALDI MSI and immunofluorescence microscopy using a pPDH antibody and DAPI staining. The cryosections used for immunofluorescence were 5 µm in thickness. Fluorescent microscopy images were acquired using a 40× objective (Zeiss Observer Z.1, Oberkochen, Germany), a DAPI filter (Filter Set 49, Carl Zeiss Microscopy, Oberkochen, Germany), and an FITC filter (31001, Chroma Technology Corporation, Bellows Falls, VT).

### MALDI matrix preparation and application

A 4.4 mg/mL of 1,5-diaminonapthalene hydrochloride (CAS: 2243-62-41, Sigma-Aldrich, Darnstadt, Germany) was dissolved in 4/4.5/0.5 HPLC grade water/ethanol/1 M HCl (v/v/v). The 10 µm thick tissue sections were sprayed using a TM-sprayer (HTX Technologies, Chapel Hill, NC) in a four-pass method. The parameters of the matrix application set in the TM-sprayer were as follows: spray nozzle velocity (1200 mm/min), track spacing (2 mm), flow rate (0.09 mL/min), spray nozzle temperature (75 °C), and nitrogen gas pressure (10 psi).

### Metabolite measurement by MSI

Metabolites in the colorectal adenoma were imaged using a timsTOF fleX mass spectrometer (Bruker Daltonics, Billerica, MA) operated in negative ion mode in full scan mode for m/z 50–600. The instrument was tuned using a low concentration tuning mix (Agilent Technologies, Santa Clara, CA) injected into the ESI source. MALDI MSI was performed at 10 µm spatial resolution. The frequency of the laser was set to 10,000 Hz, with 1000 laser shots per 10 µm pixel. The data were visualized using SCiLS Lab software (version 2021c premium, Bruker Daltonics, Billerica, MA) with total ion count normalization. Fold change measurements were determined from clusters of pPDH+/pPDH− cells and their ion intensities for hexose-phosphate, pentose phosphate, succinate, fumarate, alpha-ketoglutarate, palmitoleic acid, and oleic acid. In each image, ten ROIs (Region of Interest) consisting of ten pixels each were selected for the analysis based on palmitoleic acid ion distribution as a positive marker for pPDH+ cell clusters, and the analysis was performed from both pPDH+ and pPDH− cell clusters. We analyzed three independent sections acquired from an APCmin/SIRT6^fl/fl^/VillinCre adenoma (ten ROIs per section).

### Cell sorting

Organoids were digested with Accutase for 10’ at 4 °C to obtain single cells. Subcutaneous tumors derived from MC38 cells were harvested and dissociated to single cells using the mouse tumor dissociation kit (Miltenyi Biotech). Cell sorting experiments were done with a Beckman Coulter MoFlo Astrios cell sorter. Single cells were first gated by SSC-height/FSC-height and singlets were selected by FSC-area/FSC-width followed by SSC-area/FSC-width. Live cells were selected by DAPI-Heigth/FSC-height and analyzed for mCherry expression (FSC-height/561-614/620-mCherry-height-log). Representative gating strategies are shown in Supplementary Fig. 9.

### Software and code

IF images were acquired using a Leica SPE confocal microscope, a Leica SP8 confocal microscope or an in house-built Olympus two-photon microscope (Multiphoton Microcopy Core, Massachusetts General Hospital), depending on the experiment. IHC and ISH images were acquired with a Olympus DP72 microscope. Metabolites in the colorectal adenoma were imaged using a timsTOF fleX mass spectrometer (Bruker Daltonics, Billerica, MA). Graphpad Prism 6 and Microsoft Excel v16.15 were used for statistical analyses throughout this study. ImageJ 1.51 u was used to process IF images. STAR was used to map RNA-sequencing data. HTseq was used to assign reads to Gencode vM9. Pathway enrichment analysis was carried out using GSEA as detailed in the experimental procedures. MSI data were visualized using SCiLS Lab software (version 2021c premium, Bruker Daltonics, Billerica, MA). Flow cytometry data was analyzed by FlowJo vX.0.7.

## Supplementary information

Supplementary Information

Peer Review File

Description of Additional Supplementary Files

Supplementary Data 1

Supplementary Data 2

## Data Availability

Source data are provided with this paper. We used the following publicly available gene expression datasets: GSE99815, GSE83394.The metabolomics data have been deposited in Metabolomics Workbench under study ID ST002072. The remaining data are available in the Article and Supplementary Information files. Source data are provided with this paper.

## Source data

Source Data
