# Supplementary material for: A non-dividing cell population with high pyruvate dehydrogenase kinase activity regulates metabolic heterogeneity and tumorigenesis in the intestine
Source: Nat Commun. 2022 Mar 21;13:1503. doi: 10.1038/s41467-022-29085-y (PMC8938512; doi:10.1038/s41467-022-29085-y)
Supplement: Supplementary file 1 — Supplementary Information [file 41467_2022_29085_MOESM1_ESM.pdf]

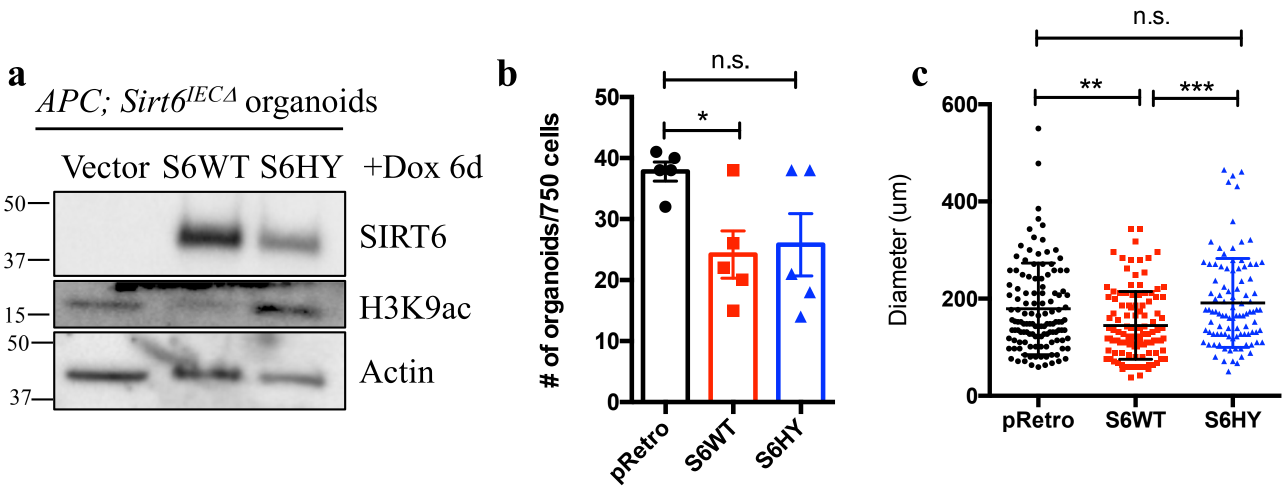

Supplementary Figure 1, related to Fig.1. SIRT6 activity regulates adenoma stem cell activity. a) Western Blot showing SIRT6 expression and H3K9ac levels in control and SIRT6 overexpressing organoids (n=1). b) Number of organoids formed by control and SIRT6-overexpressing *APC; Sirt6*$^{IEC\Delta}$ organoids. Two different experiments in triplicate are shown. Error bars indicate SEM (p=0.0117) c) Size of organoids formed by control and SIRT6-overepressing *APC; Sirt6*$^{IEC\Delta}$ organoids. Organoids from three independent experiments (each one in triplicate) are shown. Error bars indicate SD. P=0.0018 (pRetro vs S6WT) and p<0.0001 (S6WT vs S6HY). One-way ANOVA was used to determine statistical significance between groups (*, p<0.05; **, p<0.01; ***, p<0.001). Source data are provided as a Source Data file.

Supplementary Figure 1

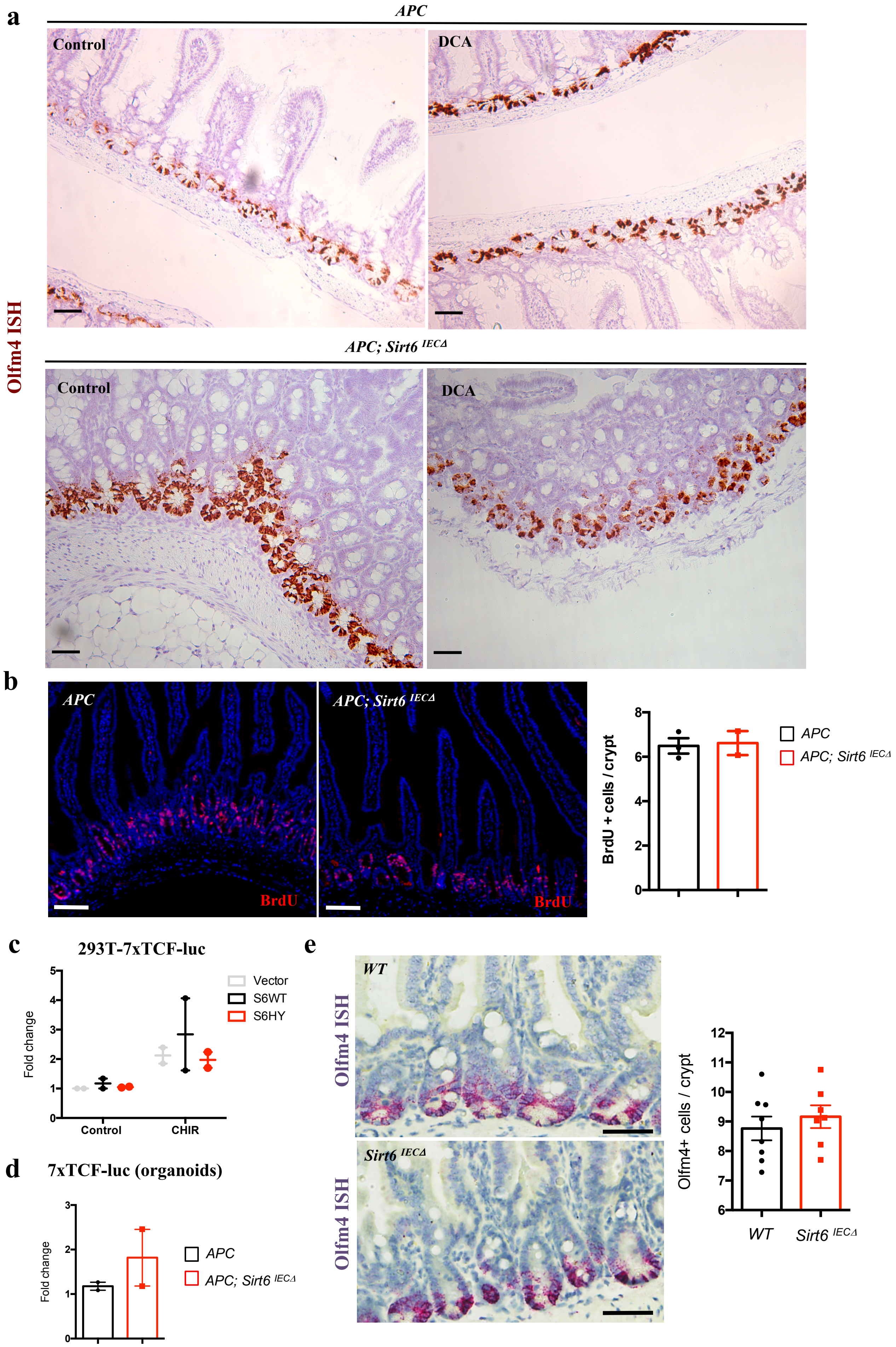

Supplementary Figure 2

Supplementary Figure 2, related to Fig.1. SIRT6-dependent ISC expansion is independent of cell proliferation and Wnt signaling. a) ISH for *Olfm4* on intestinal sections from *APC* and *APC; Sirt6*$^{IEC\Delta}$ mice treated or untreated with DCA (5g/l). Representative images of 8 *APC,* 7 *APC; Sirt6*$^{IEC\Delta}$, 5 APC+DCA and 3 *APC; Sirt6*$^{IEC\Delta}$ + DCA mice. Scale bars, 100µm. b) Immunofluorescence showing BrdU^{+} cells in the intestines of *APC* and *APC; Sirt6*$^{IEC\Delta}$ mice. Bar plot shows the number of BrdU^{+} cells in the intestines from 3 different mice. Error bars indicate SEM. Scale bars, 100µm. c) 293T cells transduced with a 7xTCF-luciferase vector were transfected with SIRT6-WT or SIRT6-HY and Wnt activity measured 48h later by the ratio luciferase/renilla. Plots represent the average of 2 different experiments (n=2) performed in triplicate (mean±SEM). d) Two *APC* and three *APC; Sirt6*$^{IEC\Delta}$ organoid lines were transduced with 7xTCF-luciferase and Wnt anctivity measured as in (c). Plot represents mean±SEM of two independent experiments done in triplicate. Two-tailed t-test was used to determine statistical significance. e) ISH for *Olfm4* on intestinal sections from control and *Sirt6*$^{IEC\Delta}$ mice. Bar plots represents the quantification of *Olfm4*$^{+}$ cells from 7 mice of each genotype. Error bars indicate SEM. Scale bars, 50µm. Source data are provided as a Source Data file.

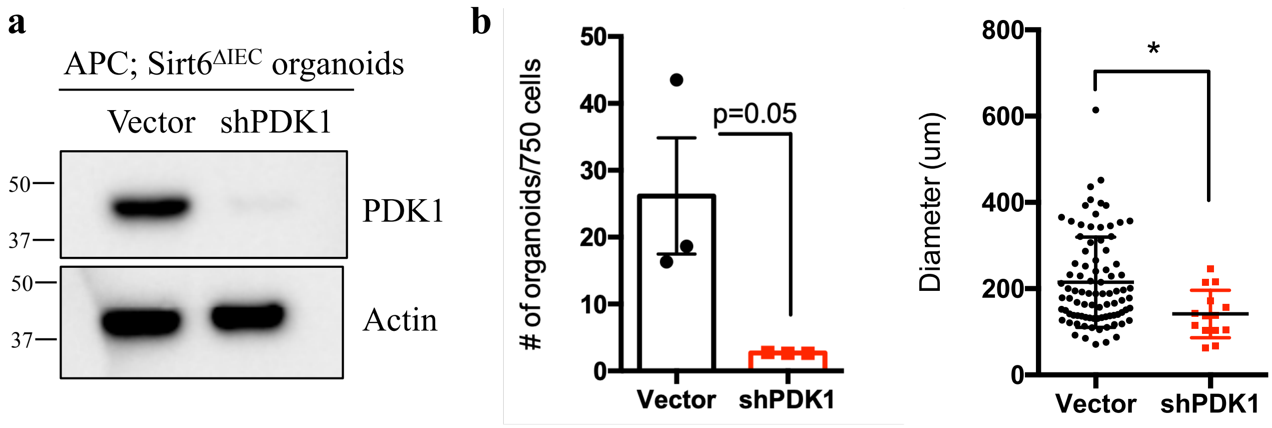

Supplementary Figure 3, related to Fig.1. Sirt6-driven tumor initiation is dependent on PDK1 activity. a) Western Blot showing PDK1 expression in control and shPDK1 organoids (representative image of two independent experiments). b) Number of organoids formed by control and shPDK1 *APC; Sirt6$^{IEC\Delta}$* organoids. Averages of three different experiments done in triplicate are shown. Error bars indicate SEM. c) Size of the organoids in b ($p=0.0115$). Error bars indicate SD. Two tailed t-test was used to determine statistical significance (*, $p<0.05$; **, $p<0.01$; ***, $p<0.001$). Source data are provided as a Source Data file.

Supplementary Figure 3

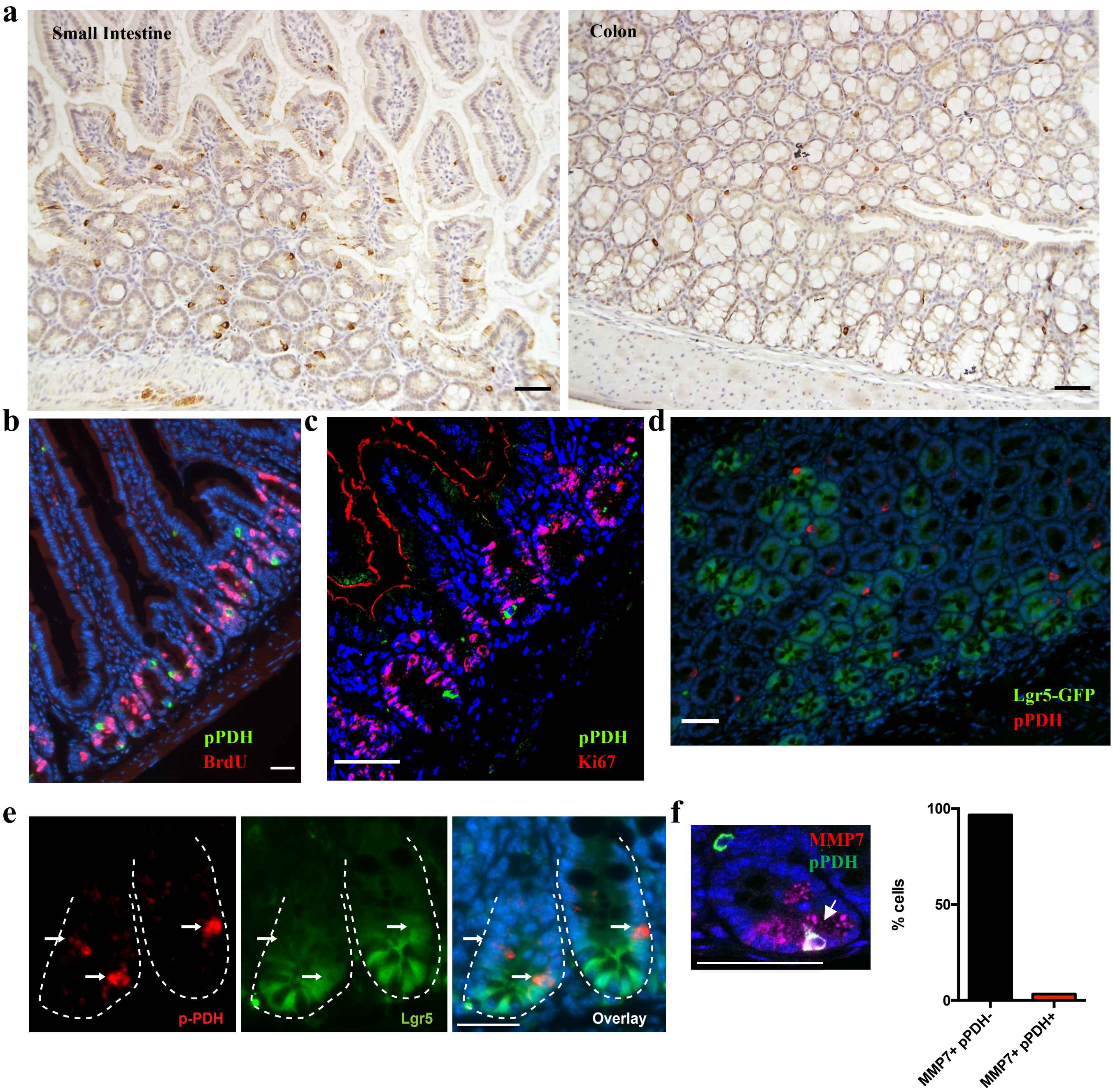

Supplementary Figure 4, related to Fig.2. pPDH is a marker of quiescent ISCs. a) Immunohistochemistry of pPDH on small intestine (left) and colon (right). This experiment was done in 4 mice and a representative image is shown. b) Immunofluorescence of pPDH and BrdU on small intestine. This experiment was done in 4 mice and a representative image is shown. c) Immunofluorescence of pPDH and Ki67 on small intestine. A representative section from the intestine of one mice is shown. d and e) Immunofluorescence of pPDH and GFP on intestinal sections from *Lgr5*$^{eGFP\text{-}IRES\text{-}CreERT2}$ mice. This experiment was done in 4 mice and representative images are shown. f) pPDH and MMP7 staining in mouse intestinal sections (left panel, arrow indicates a MMP7+pPDH+ cell) and percentage of MMP7+ Paneth cells pPDH- and pPDH+ (right panel, over 300 MMP7+ from the intestine of one mouse cells were scored). Scale bars, 50µm.

Supplementary Figure 4

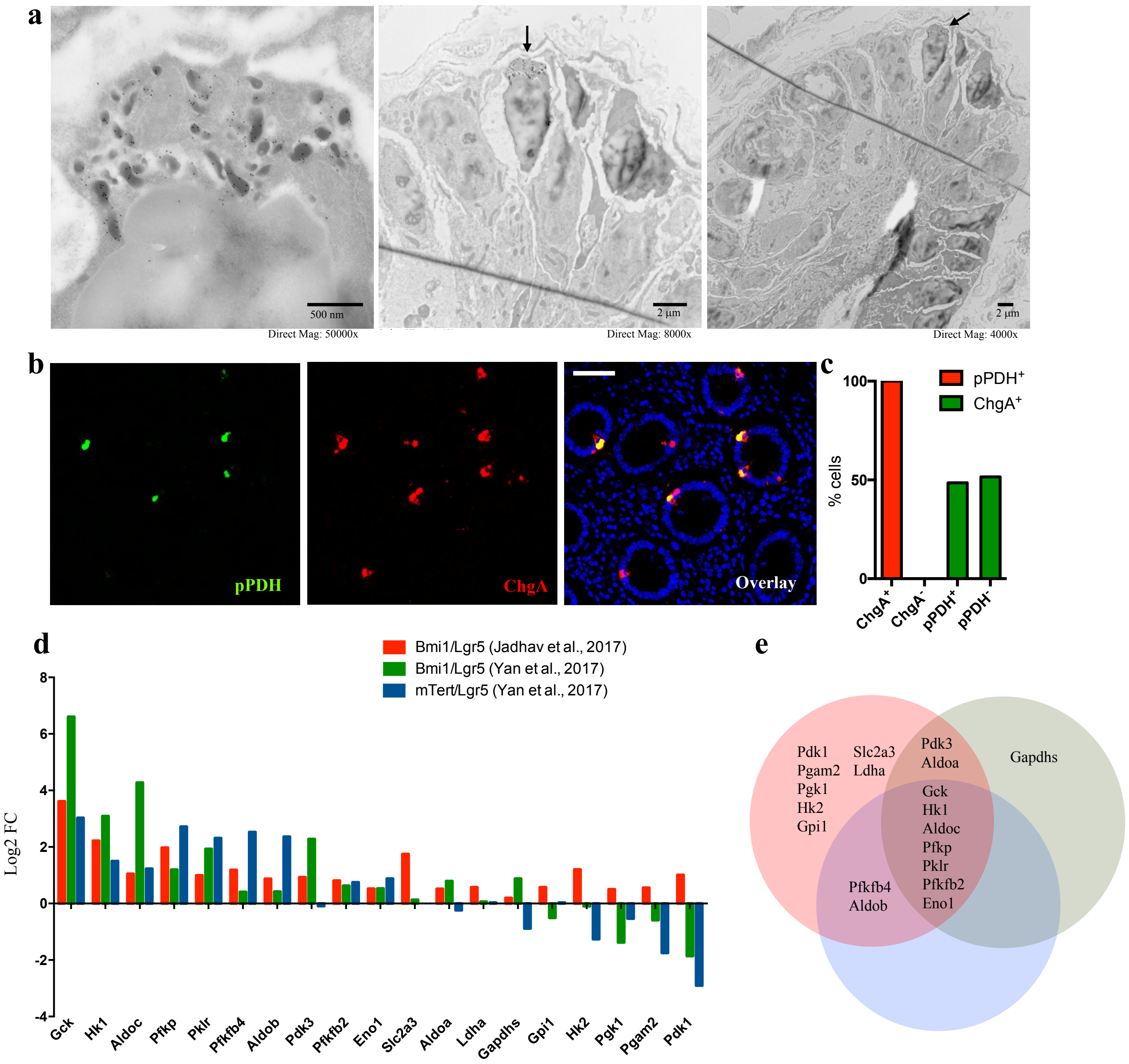

Supplementary Figure 5, related to Fig.2. pPDH is a marker of EE cells. a) Immunogold-EM experiments showing structural features (pyramid shaped cells containing secretory granules) of pPDH$^+$ cells (arrows) at 4000X (right), 8000X (middle) and 50000X (left). The experiment was performed using intestinal sections from 2 different mice (representative images are shown). b) Representative images of pPDH and ChgA staining on human small intestine (n=3 human intestinal sections). Scale bar, 50µm. c) Quantification of pPDH and ChgA positive in human small intestine (a total of 114 crypts were scored). d) Comparison of the expression (Log_2FC) of the core enriched glycolytic genes shown in Figure 2i between the datasets from Jadhav et al. and Yan et al. e) Overlapping genes from the core enriched glycolytic gene list among Bmi1$^+$ ISCs (Jadhav et al., 2017), Bmi1$^+$ ISCs (Yan et al., 2017) and mTert$^+$ ISCs (Yan et al., 2017). Source data are provided as a Source Data file.

Supplementary Figure 5

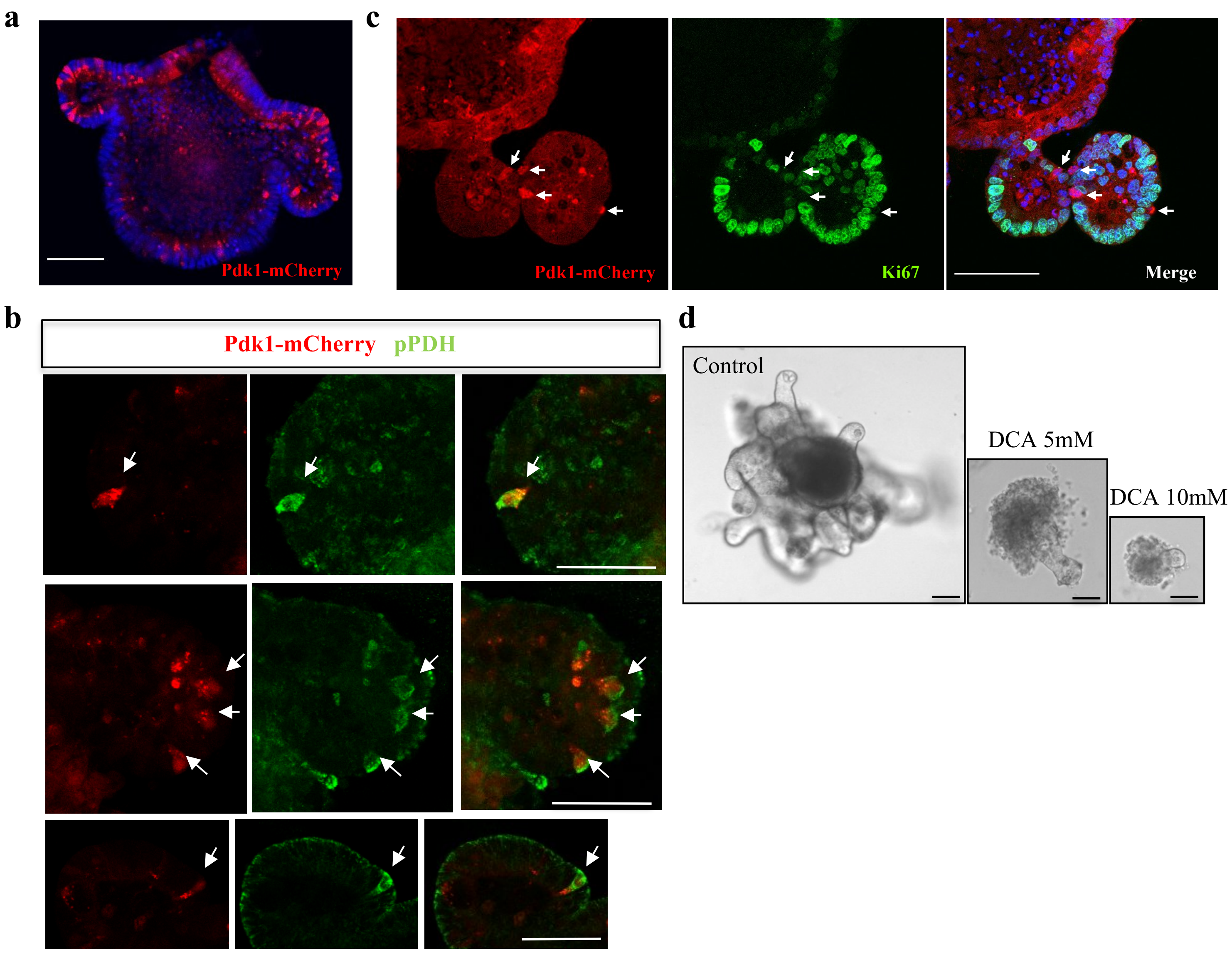

Supplementary Figure 6, related to Fig.3. Metabolic heterogeneity in intestinal organoids. a) Image of a full-grown intestinal organoid expressing the Pdk1-mCherry reporter (representative image of 10 different experiments). b) Confocal microscopy images showing that Pdk1-mCherry^{+} cells are pPDH+ (representative image of 3 independent experiments is shown). c) Confocal microscopy images showing that Pdk1-mCherry^{+} cells are Ki67^{-} (arrows). The experiment was done 4 times with similar results d) Representative images of size and shape of intestinal organoids growing in the absence or presence of DCA at the indicated concentration. Scale bars, 50µm.

Supplementary Figure 6

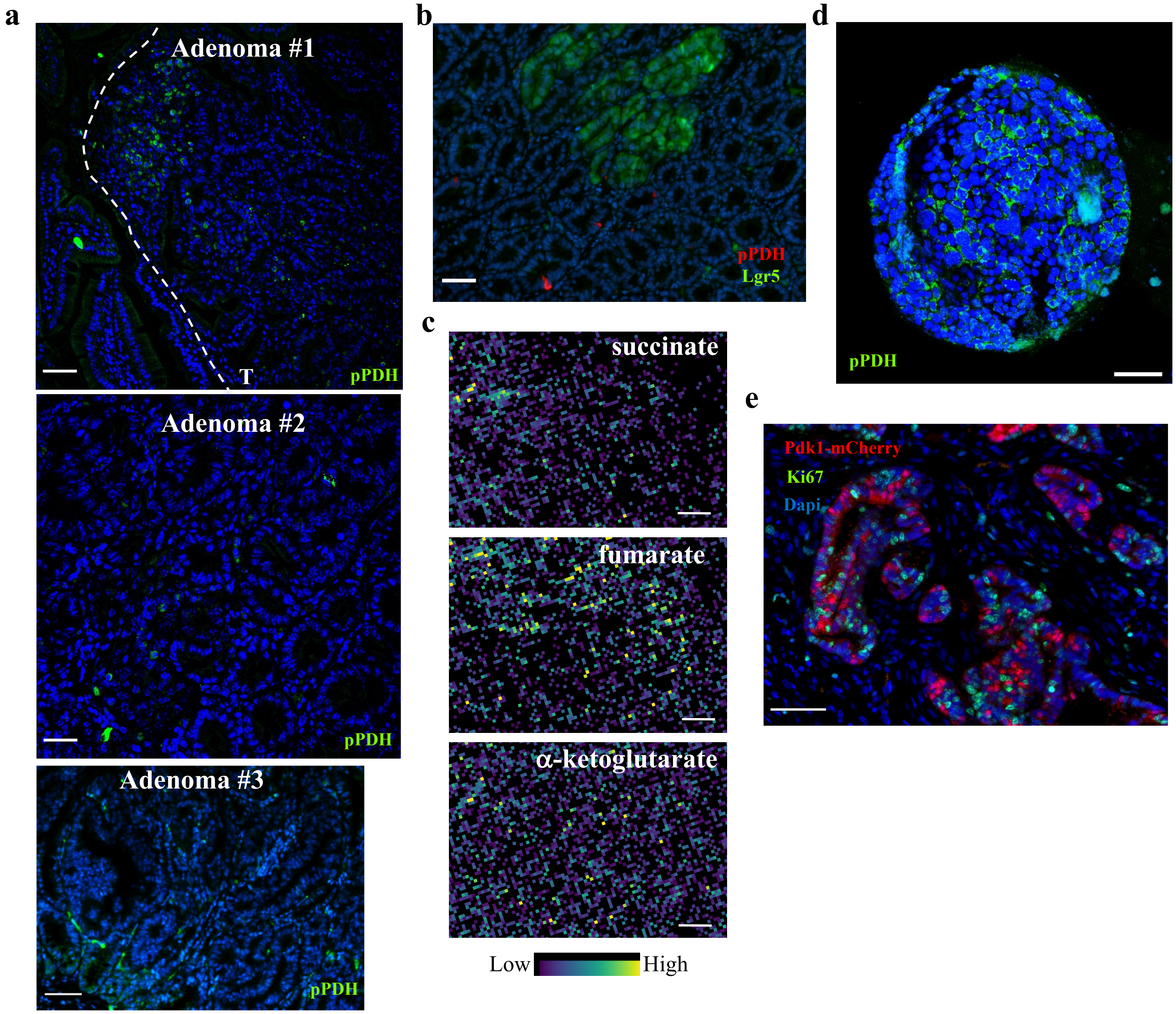

Supplementary Figure 7, related to Fig.4. Metabolic heterogeneity in intestinal adenomas. a) Immunofluorescence of pPDH in intestinal adenomas (upper panel, adenoma with several pPDH+ cells; middle panel, adenoma with few pPDH+ cells; lower panel, adenoma with no pPDH+ cells). Representative images of 24 adenomas from 6 mice are shown. Scale bars, 50µm. b) Immunofluorescence of pPDH and GFP on intestinal adenomas of an *Apc^{min}; Lgr5$^{eGFP-IRES-CreERT2}$* mouse. Representative image of adenomas from a 5 mice. Scale bars, 50µm. c) MALDI-MSI experiment showing relative abundance of indicated metabolites in the intestinal adenoma from Fig.4. Scale bars, 100µm. d) Representative image of pPDH expression in organoids derived from *Apc^{min}* adenomas. Scale bar, 50µm. e) Immunofluorescence for mCherry and Ki67 of a subcutaneous tumor grown in a SCID mouse after injection of *Apc^{min}*-derived organoids expressing the Pdk1-mCherry reporter (n=1 tumor). Scale bar, 50µm.

Supplementary Figure 7

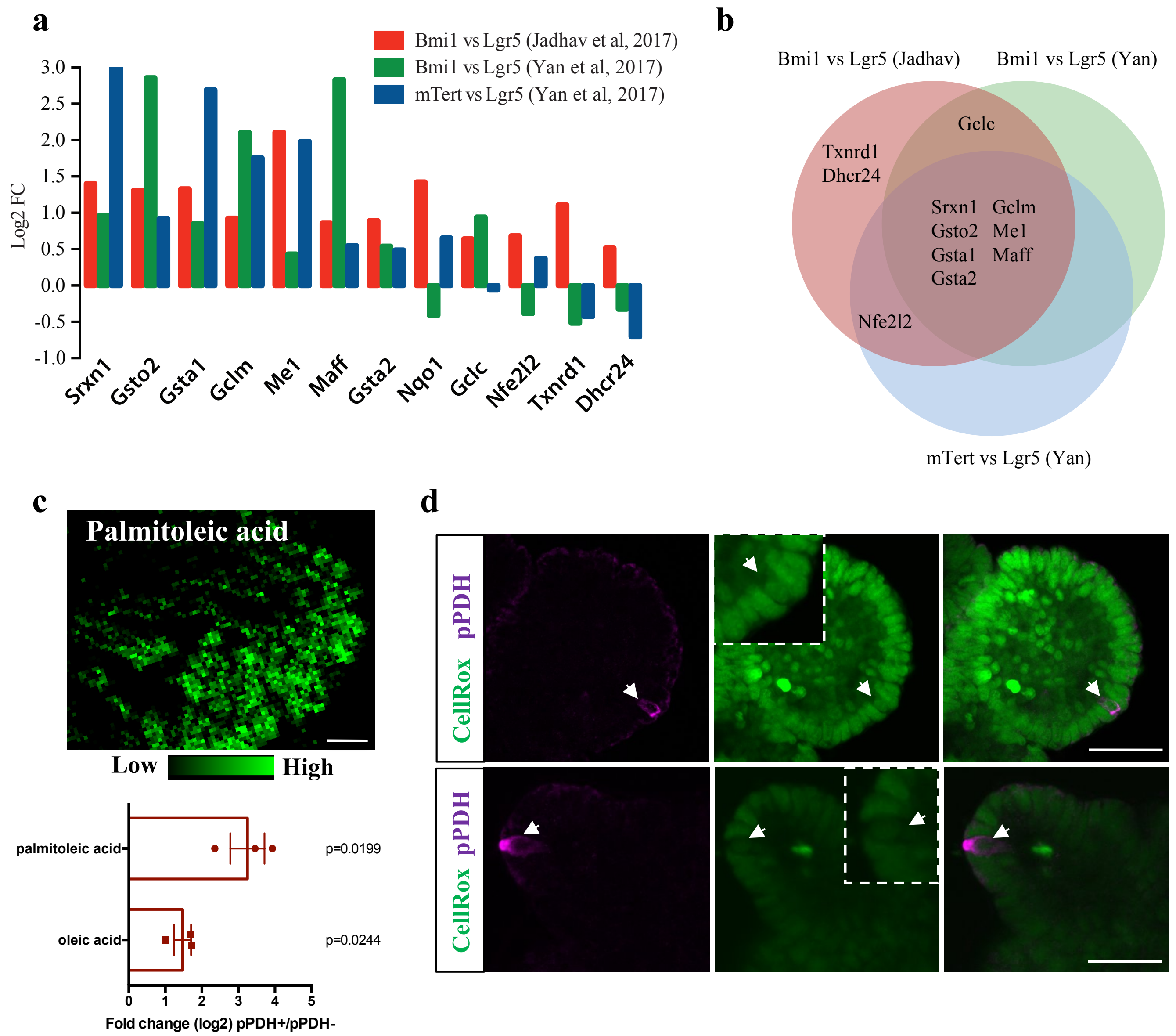

Supplementary Figure 8, related to Fig.5. Antioxidant response gene expression in +4 ISCs. a) Comparison of the expression (Log_2FC) of the core enriched antioxidant response genes between the datasets from Jadhav et al. and Yan et al. b) Overlapping genes from the core enriched antioxidant response gene list among $Bmi1^+$ ISCs (Jadhav et al., 2017), $Bmi1^+$ ISCs (Yan et al., 2017) and $mTert^+$ ISCs (Yan et al., 2017). c) MALDI-MSI experiment showing relative abundance of indicated metabolites in the intestinal adenoma from Fig.4. Lower panel represents the fold change of indicated metabolites of pPDH+ compared to pPDH- cells (p values are calculated by one sample *t-test*). n corresponds to a 100 µm (10 pixels of 10 µm size) of either pPDH+ or pPDH- clusters of cells. Data is presented as mean±SEM. Scale bar, 100µm. d) Representative images of organoids treated with CellRox-Green and stained for pPDH. Scale bars, 50µm. Source data are provided as a Source Data file.

Supplementary Figure 8

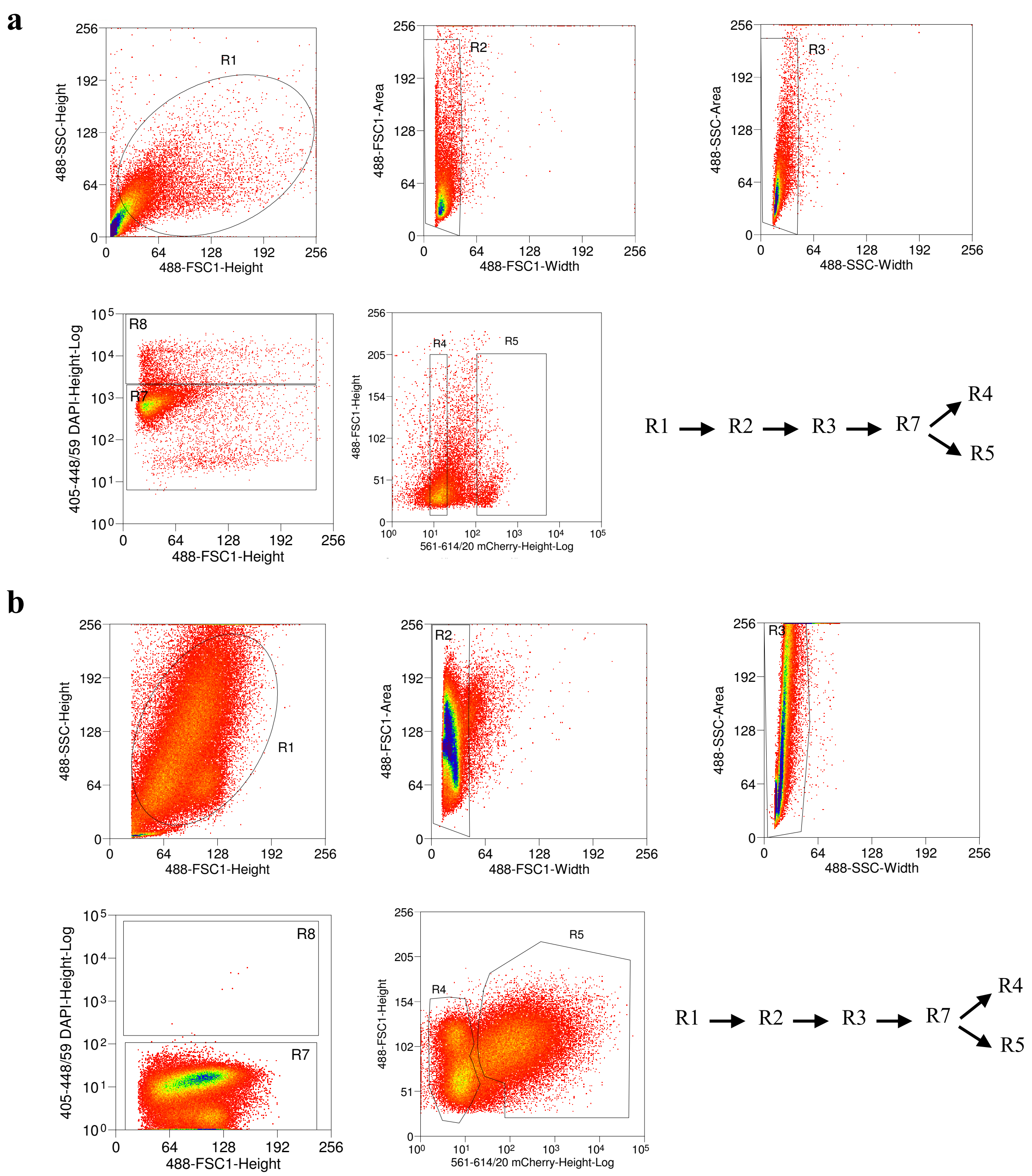

Supplementary Figure 9. Cell sorting gating strategy. a) Sorting strategy of Pdk1-mcherry organoids. b) Sorting strategy of MC38-Pdk1-mCherry tumors.

Supplementary Figure 9
