# Supplementary material for: A non-dividing cell population with high pyruvate dehydrogenase kinase activity regulates metabolic heterogeneity and tumorigenesis in the intestine
Source: Nat Commun. 2022 Mar 21;13:1503. doi: 10.1038/s41467-022-29085-y (PMC8938512; doi:10.1038/s41467-022-29085-y)
Supplement: Supplementary file 3 — Description of Additional Supplementary Files [file 41467_2022_29085_MOESM3_ESM.pdf]

Description of Additional Supplementary Files

File Name: Supplementary Data 1
Description: list of glycolytic and antioxidant genes gene-sets manually curated

File Name: Supplementary Data 2
Description: list of primer sequences used in this study
